# Molecular dissection of the glutamine synthetase-GlnR nitrogen regulatory circuitry in Gram-positive bacteria

**DOI:** 10.1038/s41467-022-31573-0

**Published:** 2022-07-01

**Authors:** Brady A. Travis, Jared V. Peck, Raul Salinas, Brandon Dopkins, Nicholas Lent, Viet D. Nguyen, Mario J. Borgnia, Richard G. Brennan, Maria A. Schumacher

**Affiliations:** 1 Department of Biochemistry, 307 Research Dr., Box 3711, Duke University Medical Center, Durham, NC 27710 USA; 2 Cryo-EM core, Department of Biochemistry and Biophysics, University of North Carolina, Chapel Hill, NC 27514 USA; 3 Genome Integrity and Structural Biology Laboratory, National Institute of Environmental Health Sciences, National Institutes of Health, Department of Health and Human Services, Research Triangle Park, NC USA

**Keywords:** Cryoelectron microscopy, X-ray crystallography, Transcriptional regulatory elements

## Abstract

How bacteria sense and respond to nitrogen levels are central questions in microbial physiology. In Gram-positive bacteria, nitrogen homeostasis is controlled by an operon encoding glutamine synthetase (GS), a dodecameric machine that assimilates ammonium into glutamine, and the GlnR repressor. GlnR detects nitrogen excess indirectly by binding glutamine-feedback-inhibited-GS (FBI-GS), which activates its transcription-repression function. The molecular mechanisms behind this regulatory circuitry, however, are unknown. Here we describe biochemical and structural analyses of GS and FBI-GS-GlnR complexes from pathogenic and non-pathogenic Gram-positive bacteria. The structures show FBI-GS binds the GlnR C-terminal domain within its active-site cavity, juxtaposing two GlnR monomers to form a DNA-binding-competent GlnR dimer. The FBI-GS-GlnR interaction stabilizes the inactive GS conformation. Strikingly, this interaction also favors a remarkable dodecamer to tetradecamer transition in some GS, breaking the paradigm that all bacterial GS are dodecamers. These data thus unveil unique structural mechanisms of transcription and enzymatic regulation.

## Abstract (web summary)

How bacteria sense and respond to nitrogen is a key question in microbial physiology. This work unveils the mechanism by which the central nitrogen metabolic enzyme, glutamine synthetase, directly signals nitrogen availability to the GlnR regulator.

## Introduction

Nitrogen is an indispensable macronutrient for all organisms. Despite its importance, it is not abundantly bioavailable, which often makes it a growth-limiting factor. Because of this, highly regulated systems for nitrogen sensing, acquisition, and utilization have evolved in most organisms^1–4^. In the cases when nitrogen levels are found to be low, there is an up-regulation of the genes encoding nitrogen acquisition factors. The same holds true when the nitrogen levels increase; the same genes should be down-regulated or repressed to conserve cellular resources. In Gram-positive bacteria, nitrogen homeostasis is not controlled by the NtrBC/σ54 systems employed by enteric Gram-negative bacteria^1–19^. In low G + C Gram-positive bacteria (Firmicutes), nitrogen homeostasis is instead controlled by the key nitrogen metabolic enzyme, glutamine synthetase (GS), and the transcriptional regulator GlnR^1,4,8–10,19–21^. In the model low G + C Gram-positive bacterium, *B. subtilis*, the transcription regulator TnrA is also involved in nitrogen regulation. However, comparative genome analyses indicate that the distribution of TnrA is limited to Bacilli^22^. By contrast, the GlnR operon (*glnRA*), encoding both GlnR and GS, is conserved among the Gram-positive bacterial clades^22^.

Early studies by Fisher et al. revealed that GS and GlnR are not only encoded in the same operon in Gram-positive bacteria but also interact directly^8,19^. This interaction is foundational to the function of GlnR as a transcriptional regulator as unlike many well-studied transcriptional regulators that sense the state of the cell by forming a complex with a small molecule metabolite, GlnR binds GS when the enzyme is in its glutamine-feedback-inhibited state (denoted FBI-GS)^8,19^. In this way GlnR senses, indirectly, excess nitrogen levels. Once bound to FBI-GS, the DNA-binding activity of GlnR is activated, allowing it to regulate the transcription of nitrogen assimilation genes^8,19^.

GS generates glutamine from glutamate, ATP, and ammonium by a mechanism that involves the initial phosphorylation of the γ-carboxyl group of glutamate by ATP and the subsequent incorporation of ammonium. Release of phosphate results in the production of glutamine^23–25^. Underscoring its fundamental role in nitrogen metabolism, GS is found in all extant life forms and phylogenetic studies indicate it is one of the oldest functional genes in living organisms^26–28^. Based on sequence, structure and mode of regulation, GS enzymes have been categorized into three different classes: GSI, GSII, and GSIII^27^. GSI and GSIII enzymes are found in bacteria and archaea and are thought to all form dodecamers^29–32^, while eukaryotes harbor GSII enzymes that were considered to exist as octamers, but more recent crystallographic data revealed a decameric oligomeric state^33–38^. GSI enzymes have been further divided into GSI-α and GSI-β subclasses^1^. GSI-β enzymes, which are found in Gram-negative bacteria, are inactivated by AMPylation (adenylation) of an active site tyrosine residue^2,3^. By contrast, GSI-α enzymes, found in Gram-positive bacteria, are not AMPylated and instead are regulated by feedback-inhibition by the product, glutamine^11^. GSI-β enzymes also differ from GSI-α proteins in that the former contains an extra ~25 amino acids not found in GSI-α proteins^24,27^.

Recent structural and biochemical analyses have started to shed light on the nitrogen regulatory system in the model Gram-positive bacterium *B. subtilis* (*Bs*)^39^. These studies revealed that *Bs* TnrA and *Bs* GlnR both have MerR-like N-terminal winged helix-turn-helix (wHTH) DNA-binding domains^40^ with C-terminal flexible tails (C-tails) that bind FBI-GS^8,16,19^. The Gram-positive actinomycetes do not encode MerR-like GlnR proteins. However, they do encode proteins, which have also been called GlnR, that belong to the OmpR/PhoB subfamily of response regulators. These latter proteins harbor folds that are distinct from the MerR-like TnrA/GlnR family^41,42^. Though TnrA and GlnR have similar structures and DNA-binding modes they are active under different conditions. TnrA is activated during conditions of nitrogen depletion and functions primarily as an activator while the DNA-binding function of GlnR is activated during nitrogen excess, and GlnR acts mainly as a repressor, notably repressing the transcription of the *glnRA* operon^4^. In addition, the flexible C-tail of GlnR autoinhibits its DNA-binding ability^19^. When the GlnR C-tail binds to FBI-GS, this autoinhibition is relieved. A low-resolution FBI-GS-TnrA C-tail structure provided insight into how TnrA binds GS, but the GS in the structure adopted a tetradecameric state^39^, which has been proposed to be a crystallization artifact^12^.

To date, the molecular mechanism by which GlnR binds FBI-GS is unknown. Underscoring the importance of understanding this conserved GS/GlnR circuitry, recent studies have shown that it contributes to virulence in several Gram-positive pathogenic bacteria, including *Listeria monocytogenes* and *Staphylococcus aureus*^10,21,43–46^. Indeed, GlnR was initially identified in *S. aureus* (*Sa*) as a factor involved in methicillin resistance (*femC*)^21^ and *Listeria monocytogenes* (*Lm*) depends on the GlnR/GS regulon for the generation of nitrogen compounds, such as glutathione and glutamine, that are important for virulence^10^. In *Paenibacillus polymyxa* (*Pp*), GlnR also plays a central role in nitrogen fixation^20^. *Sa, Lm*, and *Pp* all lack TnrA genes. Hence, GlnR is the key nitrogen regulator in these bacteria^10,20,21^. Here we show, using a combination of biochemical, crystallographic, and cryo-EM studies, the molecular mechanism by which the key nitrogen metabolic enzyme, GS, activates the activity of the GlnR regulator of nitrogen homeostasis.

## Results

### *Bs* GS is tetradecameric in *Bs* GS-TnrA and *Bs* GS-GlnR complexes

**Fig. 1:**
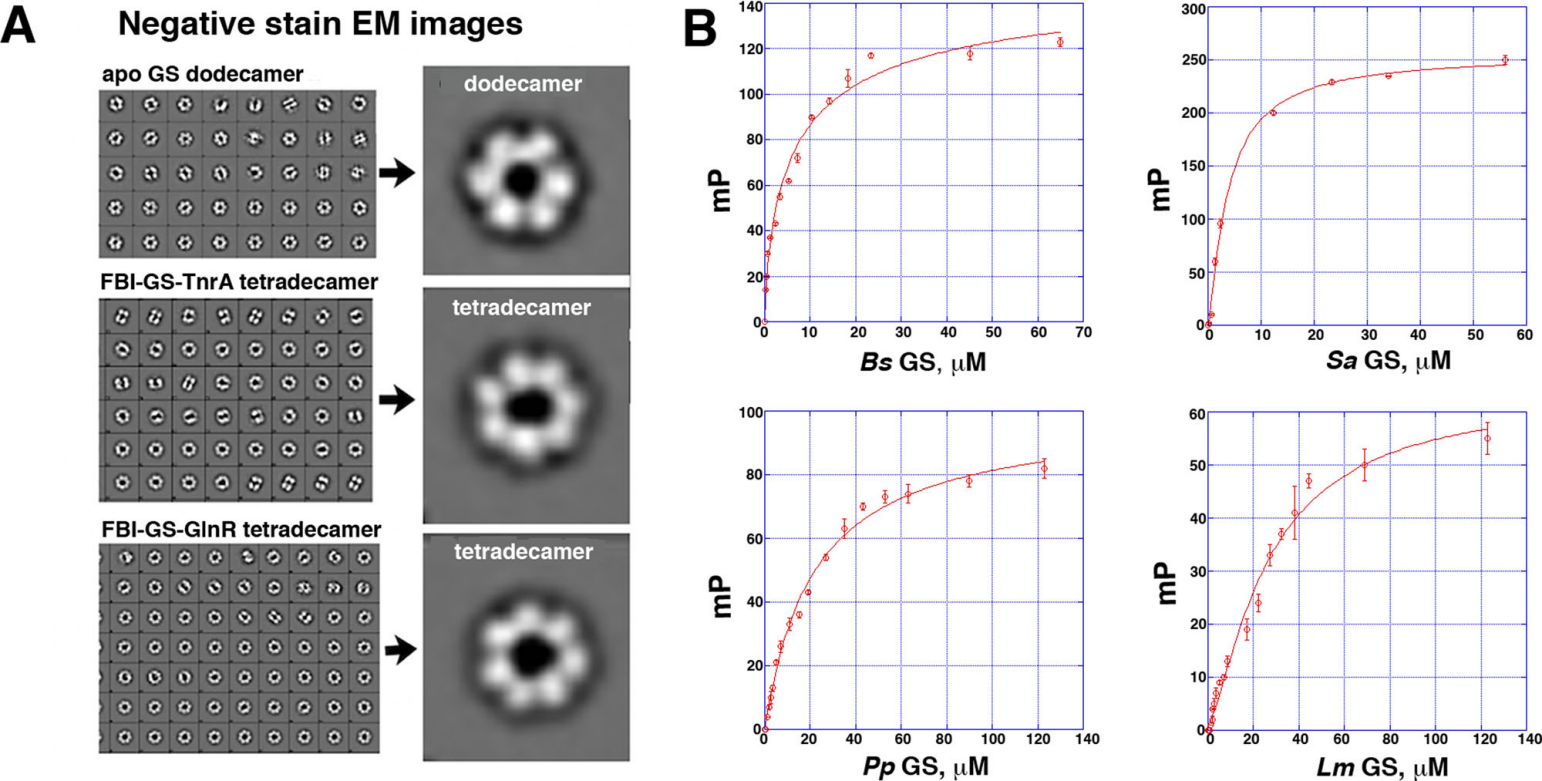
*Bs* GS forms tetradecamers in the presence of TnrA and GlnR. **A** 2D class averages of negative stain EM images of apo *Bs* Gs, *Bs* FBI-GS-TnrA, and *Bs* FBI-GS-GlnR complexes. Right shows a close-up of the top views revealing that apo *Bs* GS is a dodecamer, while *Bs* FBI-GS-TnrA and *Bs* FBI-GS-GlnR are tetradecamers. **B** Fluorescence polarization (FP) based assays analyzing *Bs* FBI-GS, *Sa* FBI-GS, *Pp* FBI-GS, and *Lm* FBI-GS binding to their corresponding fluoresceinated GlnR C-tails with K_d_s of 4.2 ± 0.3 μM, 7.2 ± 0.3 μM, 18.7 ± 3.3 μM, and 27.2 ± 3.4 μM, respectively. The curves are representative curves from three technical repeats. The error bars represent SD. Data were presented as mean values ± SD. The source data are provided in the Source Data file.

All bacterial GS structures solved to date have been dodecamers, with the exception of the *Bs* FBI-GS-TnrA C-tail crystal structure, which contains a tetradecameric GS^39^. However, this oligomeric state was postulated to be a crystallization artifact^12^. Thus, to assess the oligomeric state of *Bs* GS and the complexes it forms with TnrA and GlnR in solution we analyzed these samples by negative stain electron microscopy (EM). 2D classifications of the top views of the GS oligomers showed that apo *Bs* GS was dodecameric (Fig. 1A). Strikingly, however, not only was the GS in the FBI-GS-TnrA complex tetradecameric, consistent with the *Bs* FBI-GS-TnrA crystal structure^39^, but the GS in the FBI-GS-GlnR complex also adopted a tetradecameric state (Fig. 1A).

The *Bs* TnrA and GlnR DNA-binding domains are flexibly attached to their FBI-GS binding C-tails, indicating that these domains may not exist in a single orientation relative to the GS. Indeed, the GlnR and TnrA DNA-binding domains were not discernable in the low-resolution 2D negative stain EM classes. This finding is consistent with a recent cryo-EM study that examined fusion constructs of MBP covalently linked to GS^47^. These data revealed that only a construct with a short linker between the proteins that enabled each MBP to make the same sets of contacts with its attached GS allowed for MBP visualization^47^.

### The FBI-GS-GlnR interaction

The *Bs* GS interaction with *Bs* GlnR is arguably the best characterized. However, data suggest that *Lm, Pp*, and *Sa* GS proteins also interact with their GlnR proteins^8,19–21^. The *Bs, Lm, Pp*, and *Sa* GS proteins are highly homologous, sharing ~65% sequence identity overall. By contrast, the GlnR proteins show ~30% sequence identity, suggesting differences may exist in the GS–GlnR interaction among bacterial homologs. To begin to address this question we first quantified the FBI-GS-GlnR interaction in *Bs*, *Sa, Lm*, and *Pp* proteins by fluorescence polarization (FP) using fluorescently labeled peptides that contain GlnR C-tail residues (Methods). The *Sa, Bs, Pp*, and *Lm* FBI-GS proteins bound their corresponding fluoresceinated GlnR peptides with K_d_s of 4.2 ± 0.3 μM, 7.2 ± 0.3 μM, 18.7 ± 3.3 μM and 27.2 ± 3.4 μM, respectively (Fig. 1B).

### Structures of FBI-GS-GlnR cryo-EM complexes

**Fig. 2:**
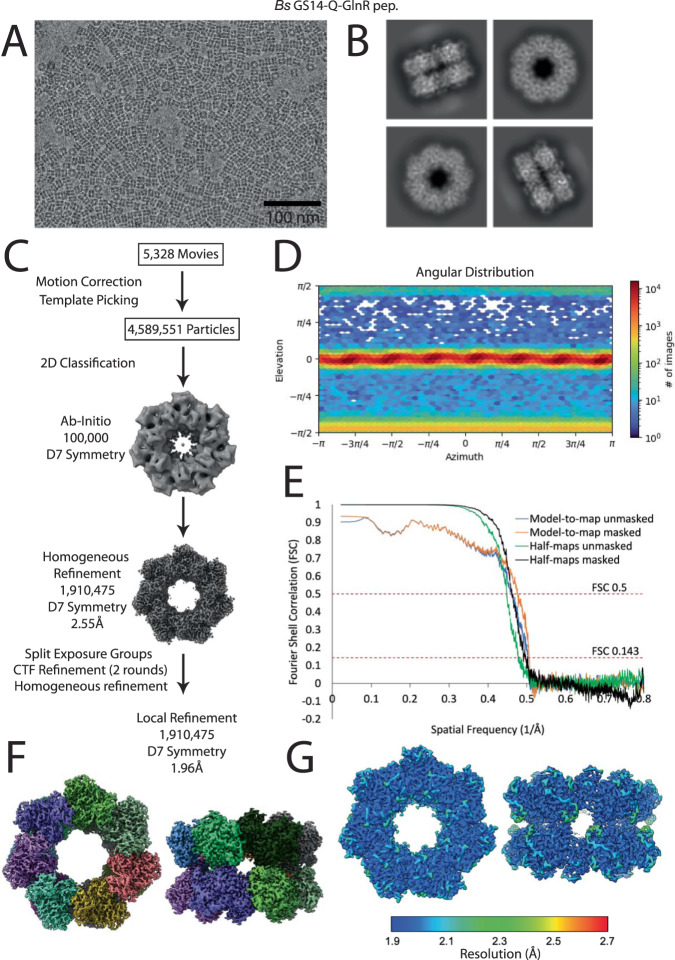
Cryo-EM data processing of the *Bs* GS14-Q-GlnR peptide dataset. **A** A representative micrograph of the *Bs* GS14-Q-GlnR peptide complex on a holey gold grid. **B** Subset of the 2D classes showing top and side views of the tetradecameric complex. **C** Summary of the data processing workflow. **D** Angular distribution plot of the final particle set. **E** Masked and unmasked half-map and model-to-map FSC curves. **F** Final sharpened map colored by GS subunit. **G** The GS structure is colored according to local resolution, with blue to red representing high to low-resolution, respectively.

**Fig. 3:**
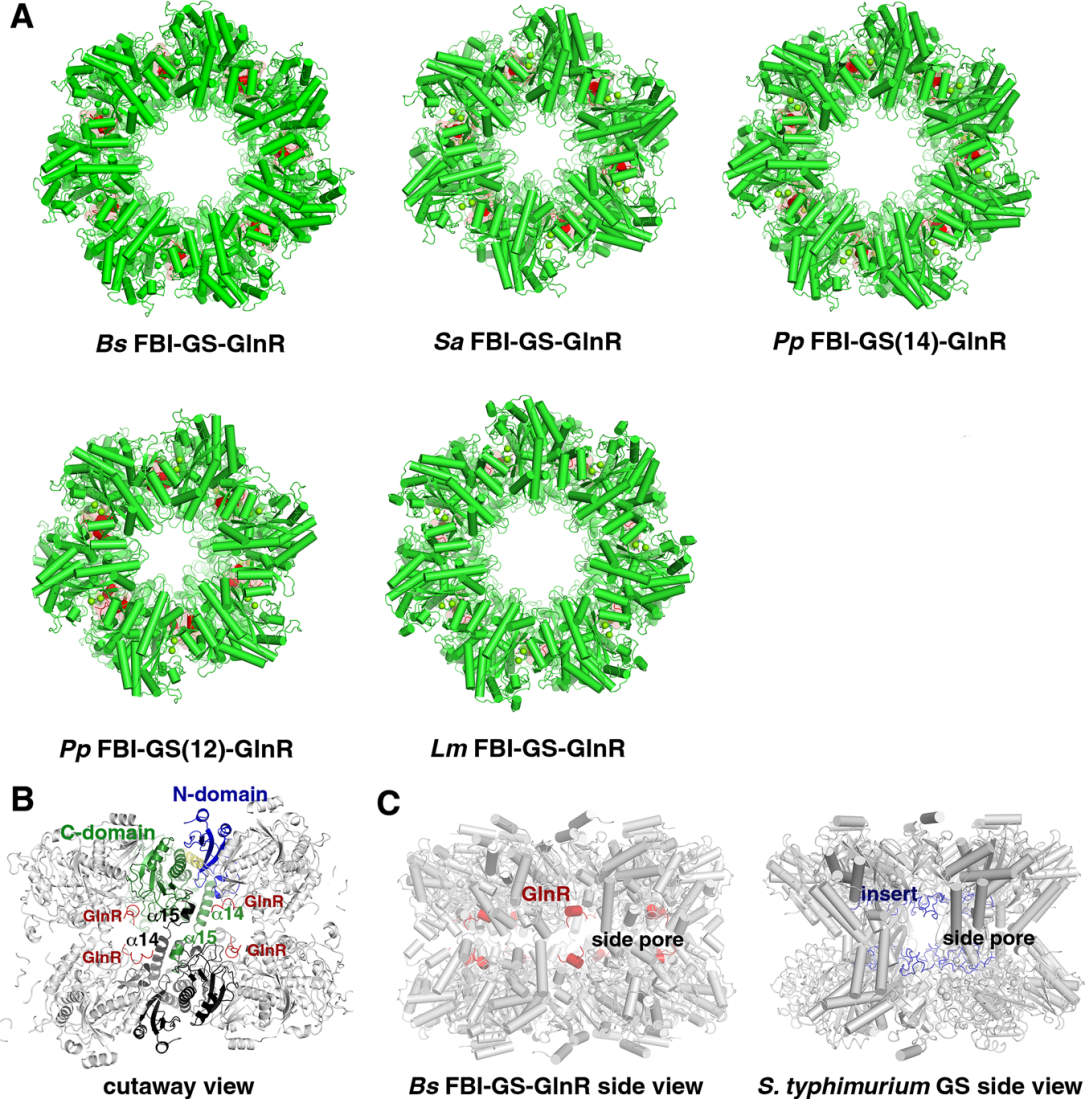
Cryo-EM structures of *Bs, Sa, Pp*, and *Lm* FBI-GS-GlnR C-tail complexes. **A** Shown are the top views of the structures for *Bs, Sa, Pp*, and *Lm* FBI-GS-GlnR C-tail complexes. GS subunits are colored green and GlnR, red. **B** Domain architecture of GS and the α14-α15 to α14″–α15″ thong interactions linking the two rings (where ″ indicates subunit from the other ring). **C** Comparison of the side view of the GlnR-bound GSI-α to the GSI-β from *S. typhimurium* showing how GlnR (red) binds in the side pore in the same location as the 25-residue insert (blue) found in GSI-β but not GSI-α.

Our negative stain EM studies showed that *Bs* GlnR binding to FBI-GS induces or stabilizes the formation of a GS tetradecamer. To ascertain if this is a conserved mechanism among FBI-GS-GlnR complexes and to elucidate, in detail, the molecular mechanism by which low G + C Gram-positive FBI-GS proteins bind GlnR, we utilized single-particle cryo-electron microscopy (cryo-EM) and solved high-resolution structures of the *Bs* (1.96 Å) (Fig. 2A–G)*, Lm* (2.61 Å)*, Pp* (2.07-2.28 Å), and *Sa* (2.15 Å) FBI-GS-GlnR C-tail complexes (Supplementary Figs. 1–4 and Supplementary Table 1). Notably, the *Bs* and *Lm* GS proteins in the complexes were all tetradecameric, while the *Pp* GS in the complex formed 10% tetradecamers and 90% dodecamers and the GS in the *Sa* FBI-GS-GlnR structure was all dodecameric (Fig. 3A).

**Fig. 4:**
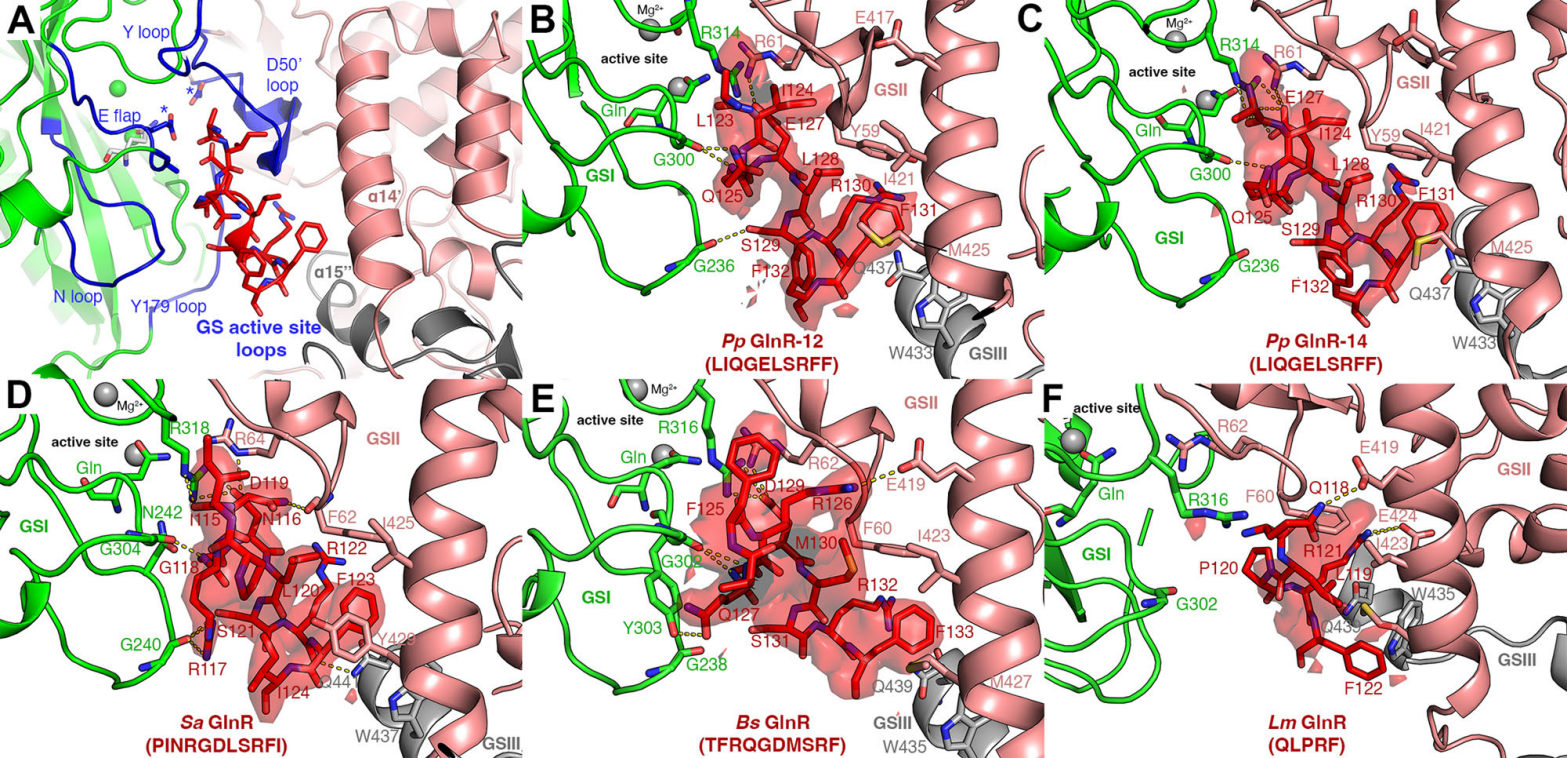
Cryo-EM FBI-GS-GlnR  structures reveal GlnR binds in the GS active site. **A** Cartoon diagram of the *Pp* GlnR C-tail (red) binding to GS. GlnR binds in the GS active site between two subunits and at the nexus of the oligomer interface between stacked rings. In this figure one GS subunit is colored green and the other salmon. The active site regions are colored blue and labeled. **B** Density for GlnR peptide and contacts with GS in the *Pp* FBI-GS(12)-GlnR complex (map contoured at 0.09 σ). Residues making contacts are labeled and different GS subunits are denoted as GSI, GSII, and GSIII, underscoring that three GS subunits participate in GlnR contacts. **C** Density for GlnR peptide and contacts with GS in the *Pp* FBI-GS(14)-GlnR complex (map contoured at 0.06 σ). **D** Density for GlnR peptide and contacts with GS in the *Sa* FBI-GS-GlnR complex (map contoured at 0.15 σ). **E** Density for GlnR peptide and contacts with GS in the *Bs* FBI-GS-GlnR complex (map contoured at 0.65 σ). **F** Density for GlnR peptide and contacts with GS in the *Lm* FBI-GS-GlnR complex (map contoured at 0.55 σ).

Despite the differences in the GS oligomeric states among these complexes, the GS subunits in each adopted essentially the same conformation (superimpositions result in root mean square deviations (rmsds) of 0.5–0.7 Å for alignment of 400 corresponding Cα atoms). Each GS subunit is composed of 15 β-strands and 15 α-helices. Helix α3 divides each subunit into a larger C-domain and a smaller, N-domain (Fig. 3B). Both GS dodecamers and tetradecamers form double-ring structures whereby the two rings are held together by interactions between C-terminal helical “thongs”, α14 and α15, with α14 and α15 from the neighboring subunit in the other ring (Fig. 3B-C). The GS active sites, which are formed at the dimer interfaces in each ring, are composed of five key regions; the E flap (*Bs* residues 300–306), the Y loop (residues 365–373), the N loop (residues 231–242), the Y^179^ loop (residues 148–158) and the D50´ loop (residues 52–66), the latter loop being the only active site region contributed from the adjacent GS subunit (Fig. 4A).

Glutamine binds identically in each structure to form FBI-GS whereby it overlaps the substrate glutamate binding site. The glutamine Nε2 atom interacts with the catalytic Glu304 residue from the E flap and the Glu304-glutamine interaction is further stabilized by a  H bond between the Glu304 side chain to Arg62´ from the D50´ loop. These contacts affix both the E flap and D50´ loop in a conformation favorable for GlnR binding. Peptide density was evident for 8-10 residues of the GlnR C-tails from *Pp* (IQGELSRF)*, Sa* (PINRGDLSRF), and *Bs* (FRQGDMSRF), and 5 GlnR C-terminal residues (QLPRF), in the *Lm* complex (Fig. 4B–F). These GlnR C-tails all form a distorted helix and bind within the GS active sites. Notably, this binding site, which is located within the GS side pores, coincides with the location of the 25 extra residue insertions found in GSI-β but not GSI-α structures (Figs. 3C and 4B–F).

### GlnR C-tail binds within the FBI-GS active site

Interestingly, the GlnR binding site in FBI-GS shows overlap with the TnrA C-tail binding site identified in our crystal structure (Supplementary Fig. 5A)^39^. This is consistent with previous data showing that mutations in *Bs* GS E flap residues 301–306 impaired interaction with both TnrA and GlnR^7^. These data were initially puzzling because the GlnR and TnrA C-tails harbor different sequences^6,17^. However, our structures show that the GlnR C-tails adopt distorted helices that make distinct contacts to GS compared to the TnrA C-tail, which forms an undistorted helix when bound to GS (Supplementary Fig. 5A, B). In the FBI-GS-GlnR structures, each GS pore binds two closely juxtaposed but non-interacting GlnR C-tails as each pore contains two active sites, one from each ring (Fig. 3C). Arguably, the most important GS active site regions are the E flap and D50´ loop; the D50´ loop provides the aspartic acid (asterisk in Fig. 4A) that abstracts a proton from ammonium while the E flap contains the catalytic glutamate (asterisk, Fig. 4A). Thus, it is striking that these GS regions provide the majority of the interactions with GlnR (Fig. 4B–F and Supplementary Fig. 6). GlnR contacts are also provided by GS residues on thong helix α14´ from the adjacent dimer and α15″ from the neighboring subunit of the other ring. Thus, the GlnR binding site within the GS active site is positioned at the nexus of the double-ring oligomerization region (Fig. 4B–F).

### A consensus GlnR binding motif for FBI–GS interaction

The FBI-GS-GlnR structures show similar overall GlnR binding modes (Fig. 4B–F). The GlnR C-tails in all but the *Lm* structure contains a glycine at the N-terminal region of the bound peptide (XG(E/D)XSRF). This glycine binds proximal to the E flap, allowing its amide nitrogen to H bond with the carbonyl oxygen of the GS glycine residue in the E flap. The corresponding residue in *Lm* GlnR is glutamine and the density corresponding to this N-terminal GlnR peptide region in the *Lm* structure is poorly resolved (Fig. 4F). Arg62´ (*Bs* numbering) from the D50´ loop interacts with an acidic residue that is generally conserved in GlnR C-tails (XG(E/D)XSRF). The GlnR acidic residue also interacts with GS residue Arg316. Arg316 is a key catalytic residue and must move to the active site during catalysis. This relocation would be prevented by GlnR binding.

The SRF motif is the most conserved region of the GlnR C-tails (Fig. 4B–F). The GlnR arginine of this motif makes contact with residues on α15´´ and stacks with the side chain of the aromatic residue in the GS D50´ loop in the *Pp, Sa*, and *Bs* structures. In the *Lm* structure this arginine contacts an α14´ acidic residue. Residues in α14´ and α15´´ also make extensive hydrophobic contact with the GlnR SRF phenylalanine residue. Finally, the serine from the SRF motif acts as a brace, anchoring the peptide across the dimer, by making an H bond to backbone atoms of E flap residues (Fig. 4B–F).

### Comparison of apo and transition state structures of *Bs, Pp, Lm*, and *Sa* GS

Previous GS structures from *E. coli, M. tuberculosis, Salmonella typhimurium*, and *Helicobacter pylori* were all dodecameric as was our apo *Bs* GS structure^24,29–32,48^. However, our EM data showed that GlnR binding results in the formation of a tetradecamer in several GS. Whether the GS tetradecamer only forms in the presence of TnrA and GlnR is unclear. Thus, we next obtained cryo-EM structures of apo *Pp, Lm*, and *Sa* GS to resolutions of 3.16, 2.85, and 2.13 Å, respectively (Supplementary Table 2). These structures revealed that, like the *Bs* apo GS, the *Lm*, *Sa*, and most of the *Pp* apo GS states are dodecamers. However, a small percentage of tetradecamers were evident in the apo *Pp* GS sample (Supplementary Fig. 7). But, due to the small number and limited side views of tetradecamers in the data, we were unable to generate a 3D reconstruction of the apo *Pp* GS tetradecamer. Nonetheless, this finding, along with our FBI-GS-GlnR structures, clearly indicates that the current paradigm that all bacterial GS enzymes are dodecamers needs revision.

Interestingly, overlays of the individual GS subunits show that the apo GS subunits are similar to those in the FBI-GS-GlnR complexes (rmsds of 0.5–0.8 Å for overlay of 400 corresponding Cα atoms between GS proteins). In this conformation, key active site residues are not properly positioned for catalysis. In particular, the Arg62 side chain is rotated into the active site preventing the proper positioning of the catalytic Asp50´ side chain and Arg316, which assists in catalysis, is moved out of the active site. Hence, conformational changes in the apo states would be needed to generate an enzymatically active conformation unless these enzymes employ a catalytic mechanism different from other GS. In fact, the electron density for the active site regions of the apo GS structures are poorly defined indicating flexibility in these regions. This suggests that they could undergo conformational changes during catalysis.

**Fig. 5:**
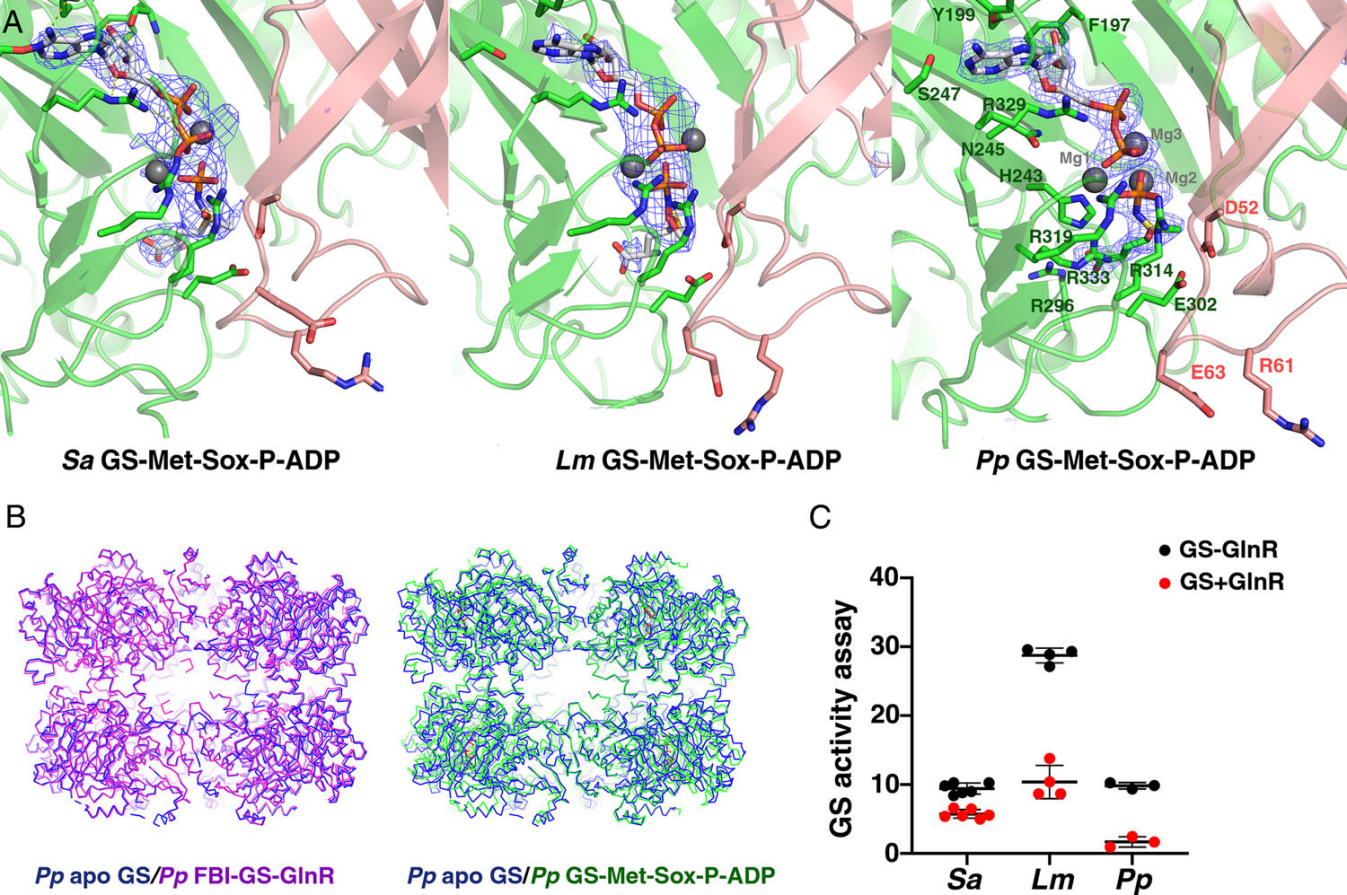
Structures of GS Met-Sox-P-ADP transition state complexes. **A** Omit electron density maps were calculated after removal of the Met-Sox-ADP and contoured at 3.8 σ for the *Sa* GS-Met-Sox-P-ADP complex, the *Lm* GS-Met-Sox-P-ADP complex, and the *Pp* GS-Met-Sox-P-ADP complex. Electron density is shown as blue mesh, GS subunits are colored green and salmon and the bound Met-Sox-P and ADP are shown as sticks. Contacts are shown and contacting residues are labeled for the high-resolution *Pp* GS-Met-Sox-P-ADP complex (the other structures show the same interactions). Interacting residues are shown as sticks and magnesium ions are shown as gray spheres. **B** Cα superimpositions of the *Pp* apo GS dodecamer (blue) onto the *Pp* FBI-GS-GlnR dodecamer (magenta) and of the *Pp* apo GS dodecamer (blue) onto the *Pp* GS-Met-Sox-P-ADP structure (green) underscoring that the apo and GlnR bound structures are similar whereas the GS-Met-Sox-P-ADP shows large differences not only at the level of subunits but overall oligomer conformation. **C** GS enzymatic assay testing the effect of excess GlnR on GS activity. For these assays, the *Sa, Lm*, and *Pp* GS and *Sa, Lm*, and *Pp* GlnR full-length proteins were utilized. GS activity was measured (as described in methods) in the presence and absence of GlnR. The results are the average of multiple measurements (from three to six) with the error bar representing SD. Two-way ANOVA using the software GraphPad Prism 9 was performed. The *P* value for all the source of variation (interaction, row factor, and column factor—as specified in the software) are statistically significant (<0.0001) in all three cases (*Lm, Sa*, and *Bs*). Source data are provided in the Source Data file.

To visualize the active, transition state (TS) conformations of the GS proteins we reacted them with l-methionine-S-sulfoximine (MSO) in the presence of ATP and solved the structures. In GS proteins this reaction leads to the formation of a stable TS analog, l-methionine-S-sulfoximine phosphate (Met-Sox-P), and ADP^32,48^. In these structures, the Met-Sox-P methyl group occupies the ammonium substrate binding site, thus mimicking the GS transition state and preventing further reaction. Structures of the *Pp, Lm*, and *Sa* GS-Met-Sox-P-ADP complexes were obtained to 1.98, 3.50, and 2.95 Å resolution, respectively (Fig. 5A and Supplementary Table 3). These structures were all dodecameric and densities for Met-Sox-P and ADP were clearly observed in each enzyme (Fig. 5A). The subunit and oligomeric structures of each TS structure were also the same and were similar to our *Bs* TS structure^32^ (rmsds of 0.5–0.7 Å for overlay of corresponding 420 Cα atoms in each subunit) (Fig. 5B). The GS TS structures all display the same contacts to the Met-Sox-P and ADP. Hence, the contacts in the *Pp* structure will be described here due to its higher resolution (Fig. 5A, right panel). In the complexes, the ADP adenine N6 is read by the carbonyl oxygen of Leu326. The side chains of Tyr199 and Arg329 make stacking interactions with the adenine base and Ser247 makes a hydrogen bond to the adenine N1 atom. Contacts to the Met-Sox-P phosphate are provided by Mg^2+^ ions and the side chains of GS residues Arg314, Arg329, Arg333, Arg334, and His243. The Arg296 side chain interacts with the Met-Sox carboxyl moiety and the Met-Sox-P amide nitrogen interacts with the Glu132 side chain as well as the carbonyl of Gly239. Catalytic E flap residue Glu302 contacts Asp52´ and is close to the Met-Sox-P methyl group. The Glu302–Asp52´ interaction would shield the Met-Sox-P from attack by bulk solvent as well as facilitate proton abstraction of the ammonium to form ammonia. The resultant ammonia would then be in an ideal position to attack the γ-glutamyl phosphate intermediate. Thus, the GSI-α·Met-Sox-P·ADP complexes mimic the tetrahedral adduct, formed before the generation of the glutamine and inorganic phosphate products.

### Structures indicate a conserved two-state model for low G + C Gram-positive GS

**Fig. 6:**
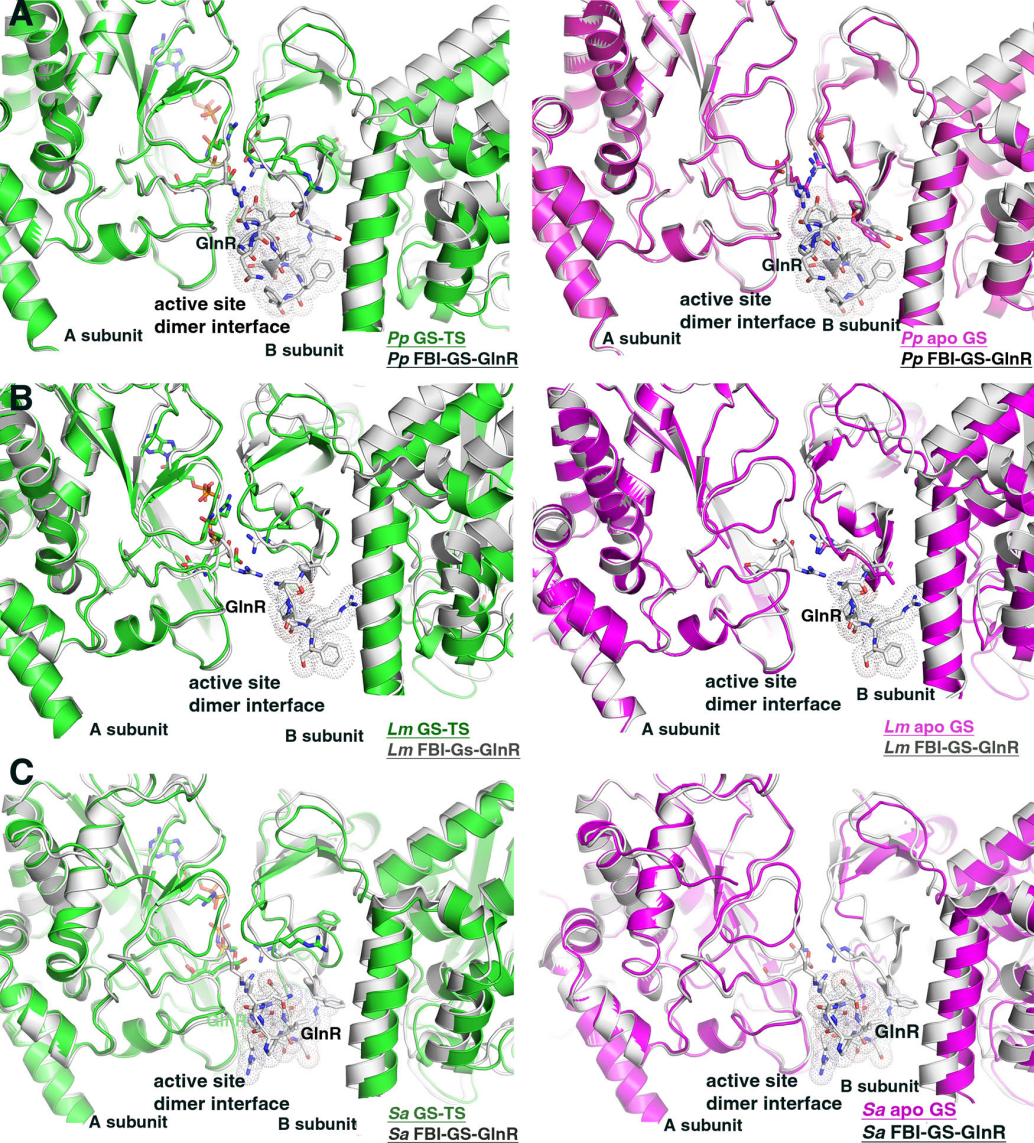
A two state model for low G+C Gram-positive GS. Views comparing the active sites of the FBI-GS-GlnR structures to their corresponding TS (left) and apo (right) states for (**A**) *Pp* GS, (**B**) *Lm* GS, (**C**) *Sa* GS. The overlays reveal that the active sites are similar in the apo and GlnR-bound states but that the Met-Sox-P-ADP bound forms are strikingly different. In these figures the apo states are colored magenta, the GlnR-bound states are gray and the Met-Sox-P-ADP (TS) bound states are green. The GlnR C-tails are shown as sticks with dotted surfaces for clarity.

Strikingly, while the TS structures are similar to each other, they show significant differences, both global and local, when compared to the corresponding apo and FBI-GS-GlnR bound states (as apo and FBI-GS-GlnR conformations are the same, the state will be referred to as apo/FBI-GS-GlnR) (Fig. 5B). The largest local structural changes between the GS TS and apo GS/FBI-GS-GlnR-bound states are within the active site loop regions (rmsd of apo GS/FBI-GS-GlnR versus Met-Sox-bound GS subunits is 2.0–2.5 Å for 400 corresponding Cα atoms), but the rmsds are 0.8 Å when the active site loops are not included. Moreover, superimposition of the TS dodecamer onto the apo GS or FBI-GS-GlnR bound oligomers (comparing Cα atoms of the dodecamers) results in rmsds of >3.0 Å, indicating that the catalytically induced structural changes are transmitted between subunits, causing significant alterations in the oligomeric structure (Fig. 5B). Although all GS active site loops are altered upon TS formation, residues in the D50´ loop undergo the most dramatic structural rearrangements (Fig. 6A–C). In the TS state, Asp62´ and Arg316 are repositioned to provide an optimal active site architecture. Thus, our structural analyses indicate that GS enzymes from low G + C Gram-positive bacteria adopt two distinct states, the active, TS conformation (herein termed the “A” state) and the conformation adopted by apo GS/FBI-GS-GlnR, termed the “I” or inhibited state. This is distinct from GSI-β enzymes the structures of which have revealed moderate active site loop movements between TS and apo states and while taut and relaxed designations for GSI-β structures have been described, they involve structural changes in loops caused by metal binding^48^.

### High GlnR concentrations can inhibit GS

The structural comparisons show that GlnR binding favors an inhibited conformation, which is also adopted by the apo state. This further suggested that at high concentrations GlnR may bind, albeit weakly, to the apo state and possibly inhibit GS activity. Hence, we performed enzyme assays to test this hypothesis and found that indeed, the addition of high concentrations of GlnR had an inhibitory effect on GS activity (Fig. 5C). Thus, the GS-GlnR interaction may be used as a possible route in the design of antimicrobials.

As noted, although the apo GS structures are not optimally configured for catalysis, their active site regions are not confined and appear flexible. Hence, these regions could readily transit to the active conformation during substrate binding and catalysis. Glutamine binding, however, stabilizes the I state. But the formation of the FBI-GS state depends on the intracellular concentrations of glutamine, which have been measured to range from 0.3 to 3 mM^49^. Thus, in the absence of GlnR, the effectiveness of glutamine inhibition would depend on the GS-glutamine binding affinity. Our structures show that *Pp, Bs, Lm*, and *Sa* GS bind glutamine identically. Thus, we used ITC and measured a K_d_ of 0.5 mM for glutamine binding to *Sa* GS (Supplementary Fig. 8). This relatively weak affinity suggests that glutamine might dissociate when its intracellular concentrations are low. The addition of GlnR would lock in the inhibited state. Thus, our combined data indicate that GlnR functions as a negative allosteric regulator of GS, possibly providing another level of regulation during conditions of nitrogen excess.

### Structure and function analyses of DNA binding by GlnR homologs

**Fig. 7:**
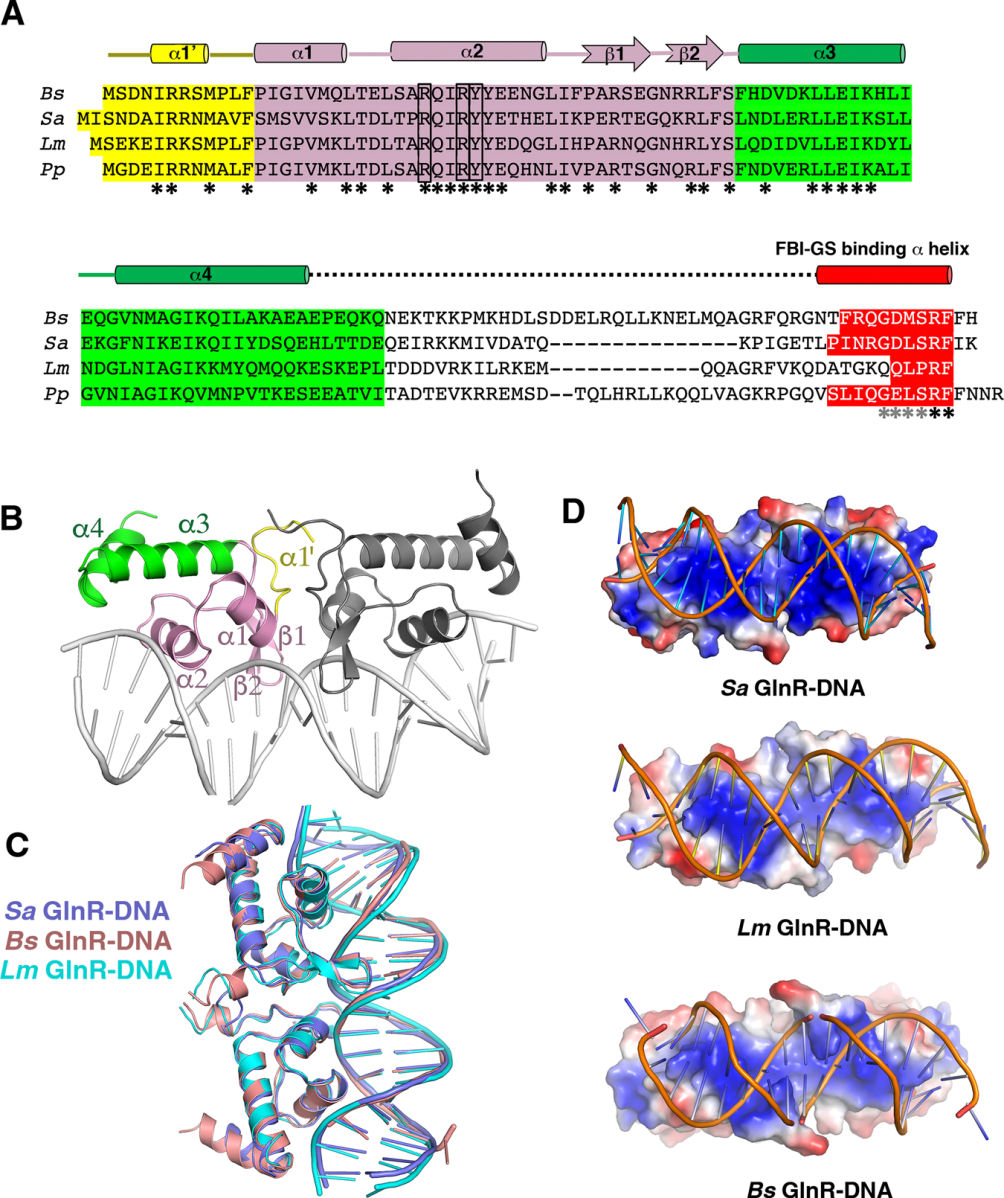
GlnR–DNA structures. **A** Multiple sequence alignment of the *Bs, Sa, Lm*, and *Pp* GlnR proteins with secondary structural elements (from *Bs, Sa*, and *Lm*) shown above the alignments. Residues in the N-terminal domains are highlighted in yellow, those in the wHTH are in pink and those in the C-terminal region are green. C-tail residues that bind FBI-GS are highlighted in red. Residues not observed in either the GlnR–DNA or FBI-GS-GlnR structures are not highlighted and form the flexible linker connecting domains. Secondary structural elements are indicated over the sequence alignments. **B** Structure of the *Sa* GlnR–DNA complex, with one subunit colored as in A and the other colored gray. **C** Superimpositions of the *Sa, Bs*, and *Lm* GlnR–DNA complexes, which are colored slate, salmon and cyan, respectively. **D** Electrostatic surface diagrams of *Sa, Bs*, and *Lm* GlnR proteins bound to DNA. Blue and red represent electropositive and electronegative surfaces, respectively. The figure was generated as a charged smoothed potential in PyMOL (*Sa* GlnR-DNA charge potentials levels range from −86 to +86, *Bs* GlnR-DNA from −73.7 to +73.7, and the *Lm* GlnR-DNA from −81.3 to +81.3).

Our FBI-GS-GlnR C-tail structures reveal the mode of GlnR binding to GS. However, to gain insight into the possible conservation of the DNA-binding mechanism among GlnR homologs and how FBI-GS binding to GlnR activates its DNA-binding function, we performed biochemical and structural analyses on GlnR–DNA interactions. Our previous *Bs* GlnR–DNA structure shows that *Bs* GlnR binds a palindromic DNA site as a dimer. Sequence homology among GlnR proteins exists primarily within the DNA-binding domain and, to a lesser extent, the last ~10 residues comprising the GS-binding domain (Fig. 7A). The linker region connecting these domains varies in both sequence and length but is expected to play a key role in GlnR function as its length and conformation would impose restrictions on the ability of GlnR DNA-binding domains, when bound to FBI-GS, to form a DNA-binding active state. The 122 residue *Sa* and *Lm* GlnR proteins have shorter linkers than *Pp* and *Bs* GlnR, which have 135 and 137 residues, respectively, and hence may not function the same in enabling DNA binding and autoinhibition as the longer GlnRs.

**Fig. 8:**
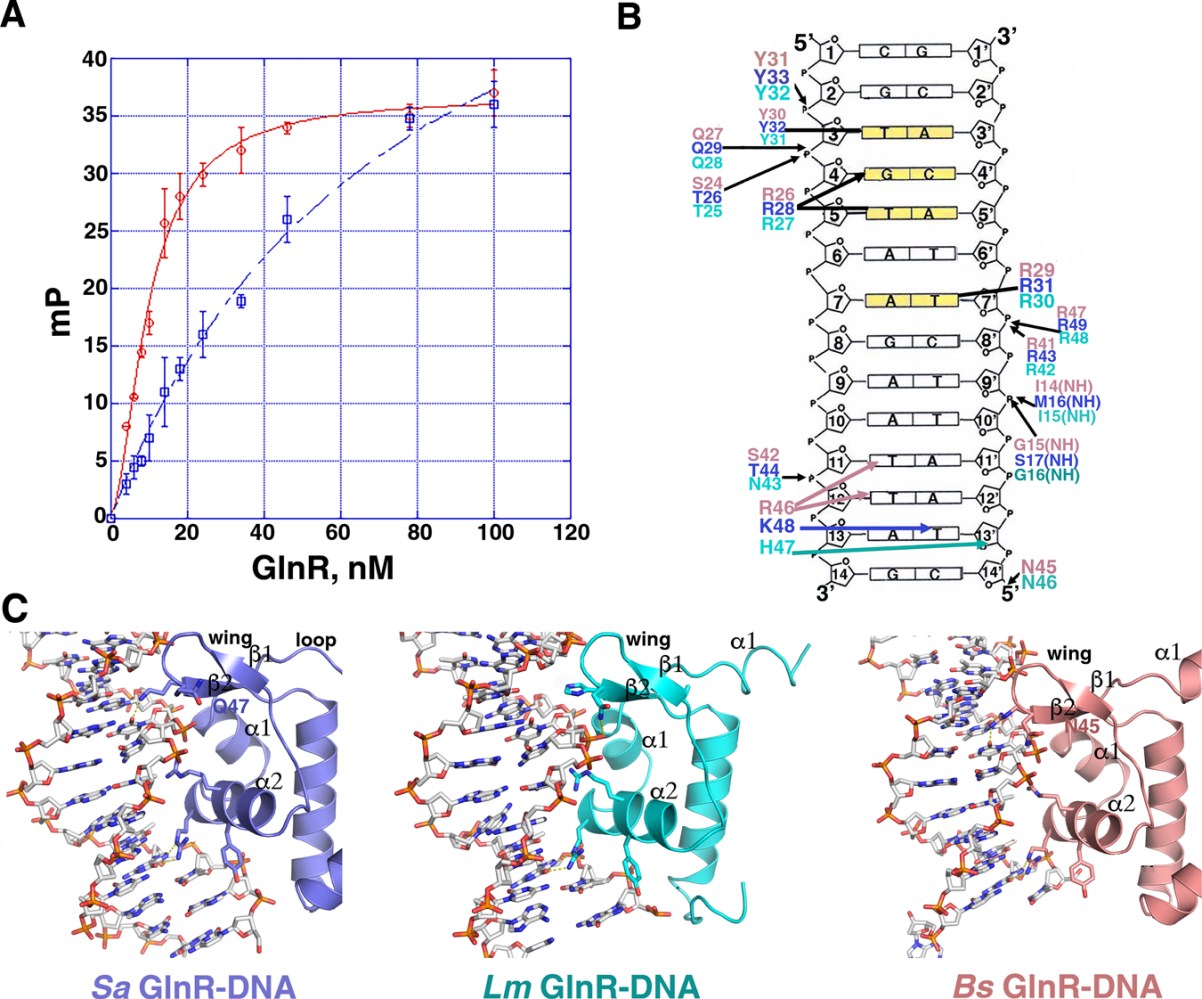
GlnR-DNA contacts. **A** FP based assays analyzing binding to the 21 bp operator by FL and truncated *Sa* GlnR, which resulted in K_d_s of 9.9 ± 0.9 nM and 75 ± 8.0 nM, respectively. The curves are representative curves from three technical repeats. The error bars represent SD. Data were presented as mean values ± SD. Source data are provided in the Source Data file. **B** Schematic representation of *Sa, Bs*, and *Lm* GlnR contacts to DNA. Residues that contact the DNA from *Sa, Bs*, and *Lm* GlnR are indicated in salmon, cyan, and slate, respectively. **C** Close-up view of the DNA contacts from each GlnR subunit to a half-site (contacts by the other subunit to the other half-site are identical).

Thus, to determine if the C-tail of the shorter *Sa* GlnR is autoinhibitory, as observed for the *Bs* GlnR, we measured DNA binding by FL *Sa* GlnR and truncated *Sa* GlnR, the latter protein lacking C-tail residues (Methods). These experiments, which revealed K_d_s of 9.9 ± 0.9 nM and 75 ± 8.0 nM for the truncated and FL *Sa* GlnR protein, respectively, support the presence of the *Sa* GlnR C-tail inhibits DNA binding (Fig. 8A). We also showed that a fluoresceinated *Sa* GlnR C-tail peptide binds to truncated *Sa* GlnR, providing support for direct autoinhibition by the GlnR C-tail (Supplementary Fig. 9).

### *Lm* and *Sa* GlnR-DNA structures

We next obtained structures of *Sa* GlnR and *Lm* GlnR bound to a 21 bp *glnR* consensus operator site to 2.35 and 3.45 Å, respectively, by X-ray crystallography (Methods; Supplementary Table 4). The GlnR structures are similar to each other and the *Bs* GlnR (rmsd = 0.6–0.8 Å for overlay of 67 corresponding Cα atoms). Each GlnR subunit can be divided into three regions, a variable N-terminal region, a MerR-like^39,40^ winged-HTH (with topology: α1-α2-β1-β2), and a short helical domain (Fig. 7A-B). The latter region forms two α-helices, α3-α4, in the *Sa* GlnR-DNA, but in the *Lm* GlnR-DNA structure, this region contains just one helix (Fig. 7C). Residues 2–73 and 2–83 were visible for the *Lm* and *Sa* GlnR proteins respectively. The *Lm* and *Sa* GlnRs form comparable dimers to the *Bs* GlnR and dock on the DNA similarly (Fig. 7C). As observed in the *Bs* structure, both DNA sites bound by *Sa* and *Lm* GlnR are bent inward but the DNA from the *Lm* complex is slightly less bent (~21°) compared to the *Sa* and *Bs* structures (~25°) (Fig. 7C). However, like *Bs* GlnR, the *Sa* and *Lm* GlnR surfaces that contact the DNA are both electropositive, indicating electrostatics as a general contributor to GlnR DNA binding (Fig. 7D).

### GlnR-DNA operator contacts

The GlnR DNA used for crystallization contains four conserved bps (bolded) in each half-site, 5′-CG**TGT**C**A**GATAATC**T**G**ACA**CG-3′ (where the consensus binding site is N_1_N_2_**T**_**3**_**G**_**4**_**T**_**5**_N_6_**A**_**7**_N(7)**T**_**7’**_N_6’_**A**_**5’**_**C**_**4’**_**A**_**3’**_N_2’_N_1’;_ N indicates any nucleotide)^4,19^. These conserved bps are contacted in the major groove by *Lm* and *Sa* GlnR residues that are completely conserved among GlnRs; a conserved tyrosine (*Sa* Tyr32/*Lm* Tyr31) makes hydrophobic contacts to the DNA thymine3 (T3) methyl group, an arginine (*Sa* Arg28/*Lm* Arg27) specifically reads the G4 base and makes hydrophobic interactions with the methyl moiety of T5 and finally, the side chain methylene carbons of a conserved arginine (*Sa* Arg31/*Lm* Arg30) contacts the T7′ methyl group (Fig. 8B-C). Previous binding studies showing that substitution of G4 to any other nucleotide abrogates GlnR binding and substitution of conserved nucleobases 3, 5, and 7 leads to significant reductions in binding are consistent with the structures^15^.

Phosphate contacts help anchor the GlnR proteins to the DNA and are mostly conserved. However, GlnR proteins contain different wing residues, which our structures show make distinct contacts to the minor groove; wing residues *Bs* Arg46 and *Sa* Lys48 make nonspecific base contacts in the minor groove, but the corresponding *Lm* GlnR residue, His47, makes hydrophobic contacts to a ribose. The lack of minor groove base contacts by *Lm* GlnR may explain the slightly reduced DNA bend observed in this structure as the minor groove base contacts by *Bs* Arg46 and *Sa* Lys48 are facilitated by the more bent DNA conformation (Fig. 8B). To test the role of this wing residue in operator DNA-binding affinity, we mutated the *Sa* GlnR Lys48 residue to arginine and histidine, which are found in *Bs* and *Lm* GlnR and performed FP studies. The K_d_s determined in these experiments were 9.9 ± 0.8 nM, 8.0 ± 0.6 nM, and 11.6 ± 0.6 nM for WT *Sa* GlnR(1-87), *Sa* GlnR(1-87)K48R, and *Sa* GlnR(1-87)K48H, respectively (Supplementary Fig. 10; Methods). Thus, these data indicate that the different wing contacts by GlnR proteins do not significantly impact operator binding affinity.

### Mechanism for GS mediated DNA-binding activation of GlnR

**Fig. 9:**
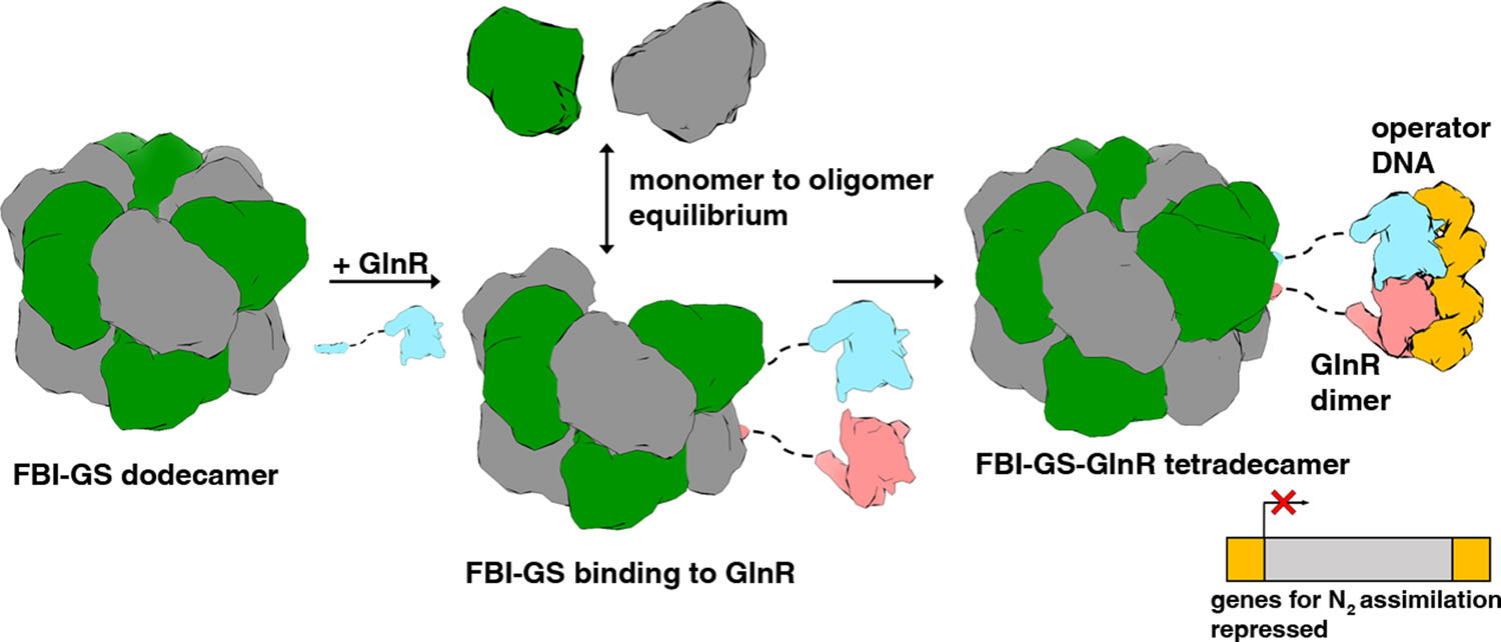
Schematic showing a structure-based mechanism of FBI-GS activation of GlnR proteins in Gram-positive bacteria. GS and GlnR are shown as schematic surface renderings with GS subunits colored gray and green and GlnR subunits colored pink and light blue. In the model GlnR binding stabilizes or facilitates subunit exchange and, in some bacteria, the transition of GS from a dodecamer to a tetradecamer. GlnR is monomeric but the interaction of two GlnR subunits in the same side pore brings them into proximity and allows them to dimerize on cognate DNA (shown as a yellow cartoon) and thus regulate genes important in nitrogen homeostasis.

A notable feature of the GlnR-DNA structures is the small GlnR dimer interface. These interfaces are formed by hydrophobic contacts between residues in the N-terminal domains. The *Sa* GlnR dimer is generated by interacting N-terminal loops, while the *Lm* and the *Bs* N-terminal regions fold into distorted helices that interact. Thus, the GlnR dimer interfaces created upon DNA binding show some variability. Nonetheless, all these interfaces bury significantly less surface area (~300–400 Å^2^ BSA) than the >2000 Å^2^ shielded by physiologically relevant dimers^50^. Indeed, most GlnR proteins have been reported to be monomeric^39^ and while a few studies suggest some GlnR proteins may dimerize or exist in an equilibrium between monomer and dimers, these experiments were typically performed at high, nonphysiologically relevant concentrations^20^. Clearly, control of GlnR dimerization and hence specific DNA binding would be an efficient mechanism of gene regulation. Thus, these data combined with our FBI-GS-GlnR structures indicate a mechanism for FBI-GS activation of GlnR binding. Specifically, our data show that the C-terminal ~10 residues of GlnR form a distorted helix when bound to FBI-GS while our GlnR-DNA structures revealed residue 84 as the last ordered/visible residue in GlnR-DNA complexes. The flexible connection between these domains would thus be minimally comprised of ~30 residues (calculated as the number of residues between the first and last residues visible in the *Sa* GS-GlnR and *Sa* GlnR structures; the *Bs* and *Lm* disordered linkers are >40 residues), which if fully extended could span >90 Å. Two C-tails bound by FBI-GS in each side pore are separated by only 7 Å, which would juxtapose the extended linkages between the domains, increasing the local concentration of the GlnR DNA-binding domains, facilitating dimer formation on operator DNA (Fig. 9).

## Discussion

Glutamine synthetases are one of the most ancient enzymes and are essential for all organisms^26–28^. Thus, these enzymes have been extensively studied. These investigations include multiple efforts to target GS function in the development of herbicides and therapeutics; plant GS are currently targeted by the herbicide glufosinate^51^ and GS enzymes have been proposed as promising drug targets in the treatment of Mycobacterial infections^48^ and cancer^52^. Despite extensive studies on GS, our work shows that much remains to be learned regarding the structural and functional aspects of these enzymes as well their regulation. Indeed, long-held dogma in the field has been that bacterial GS form dodecamers^24^. Using electron microscopy, we show here that some Gram-positive bacterial GS enzymes can exist in a tetradecameric state and that this state is favored by binding to the master transcription regulator of nitrogen metabolism, GlnR.

The FBI-GS-GlnR circuitry represents a unique regulatory paradigm in which a key metabolic enzyme directly transduces nutrient availability to the transcription regulator (GlnR). Our studies on the GS and GlnR proteins from *B. subtilis, S. aureus, P. polymyxa*, and *L. monocytogenes* show that the GS enzymes form large oligomers composed of two stacked rings, and exist in two distinct states, a state optimal for catalysis and an inactive state. However, while all bacterial GS form oligomers composed of two stacked rings, there has been no known function for this double-stacked structure and the side cavities between rings. Our high-resolution structures of *Pp, Bs, Lm*, and *Sa* GS-GlnR complexes revealed a role for this pore, which is to function as a cavity for GlnR C-tail binding. In this way, GS functions as a GlnR chaperone, facilitating the folding of the GlnR C-tail and leading to GlnR DNA-binding activation.

It is currently unclear why GlnR binding to some GS proteins favors the tetradecameric form over the dodecamer and why some GS can form tetradecamers in their apo state. However, the data point to the helical thongs, α14 and α15, as being important  in determining the oligomer state. In particular, our structures show that the deep insertion and concomitant folding of the GlnR C-tail into the central GS pore region occurs at the nexus where the two rings join, namely α14 and α15. This insertion could enhance and/or stabilize subunit exchange favoring the tetradecamer. In fact, our finding that Gram-positive GS (GSI-α) enzymes can exist in dodecameric and tetradecameric states was unusual but is consonant with data showing that GS proteins, in general, exist in an equilibrium between monomeric and oligomeric conformations. Indeed, size exclusion chromatography (SEC) analyses on GS from *E. coli*, *Ruminococcus albus 8, Neurospora crassa*, and the plant *Phaseolus vulgaris* revealed the presence in solution of both monomers and higher-order oligomers^53–56^. We have also observed monomers of GS in equilibrium with higher-order oligomers in SEC experiments on *Bs* GS^39^. Subunit exchange between oligomers would impair GS activity as the active sites are formed between two GS subunits and because GS enzymes are cooperative^32,53^ such disruption could impact the activity of the entire oligomeric complex.

Our data show that the tetradecamer form is favored in the presence of GlnR and TnrA in some GS, indicating that these regulators shift the equilibrium towards the tetradecameric state. Because GS undergoes transitions from monomeric to oligomeric states, monomer dissociation could result in an unoccupied site within each stacked layer that could be fit by the insertion of an additional GS subunit (Fig. 9). The distinct residues in the GS interfaces and/or the binding affinities of the GlnR C-tail for its GS partner may contribute to this phenomenon. In this regard, it is interesting to note that while the *Lm* GlnR peptide binds with lower affinity to its FBI-GS, the most ordered part of its C-tail is its SRF motif, which makes contacts to α14´ and α15´´. In addition, GS sequence alignments show that residues in α14 and the connection between α14 and α15 that contact GlnR and contribute to the oligomer interface show a higher degree of sequence variability than the rest of the protein and thus could determine whether a given GS can form a tetradecamer (Supplementary Fig. 6). However, testing the hypothesis that α14-α15 residues determine GS oligomeric states and can be influenced by GlnR binding is complicated by the fact that mutation of these residues may also impact GlnR binding. Thus, future studies will be required to dissect the possible roles of α14 and α15 in GS oligomer selection.

Our data show that FBI-GS-GlnR complexes not only influence the GS oligomeric state but also favor the inhibited state, providing another level of regulation to the system. Interestingly, other GS-binding polypeptides have recently been identified and characterized. For example, the *Methanosarcina mazei* GS was shown to bind a 23 residue ORF encoded peptide, called sP26^57^, the soybean GS binds the C-terminal domain of nodulin 26 (Nod26), a key symbiosome protein that is involved in nitrogen fixation^58^ and the cyanobacterial GS interacts with small proteins called IFs that inhibit its activity^59–63^. The molecular bases for these interactions are currently unknown. But these findings show that protein-protein interactions are utilized to regulate GS enzymes of prokaryotes and eukaryotes. Interestingly, while no structural information is available, data on the GS-sP26 interaction indicate that sP26 activates GS activity by shifting its equilibrium from the monomer to the higher-order oligomer state^57^. Like other GS enzymes, the *M. mazei* GS must form an oligomer for its activity as its active sites are formed between two subunits in the oligomer. These studies further underscore the importance of the GS oligomeric state for its activity.

A key function of the FBI-GS-GlnR interaction is an activation of the DNA-binding activity of GlnR. GlnR is monomeric in the absence of GS and operator DNA. Our combined data unveil a molecular mechanism for FBI-GS-mediated activation of GlnR whereby the juxtapositioning (7 Å) of two GlnR monomers bound in adjacent active sites facilitates GlnR dimer contacts on DNA. Specifically, our GlnR-DNA structures reveal that each protein has a short, ordered C-terminal region after the wHTH that is connected, via an extended linker of ~30 or more residues to the FBI-GS-binding domain. By contrast, the DNA-binding activity of TnrA, which is abrogated by its interaction with FBI-GS, has only four flexible residues connecting its FBI-GS and DNA-binding domains. Hence, even if fully extended, two TnrA subunits would be unable to interact on a DNA to form a DNA-binding dimer. Thus, the longer linker between the FBI-GS and DNA-binding regions in GlnR differentiates GlnR from TnrA and leads to the FBI–GS interaction facilitating dimer formation in GlnR rather than preventing or disrupting dimer formation.

In conclusion, our combined data provide detailed, high-resolution molecular snapshots of the nitrogen regulatory machinery from multiple non-pathogenic and pathogenic low G + C Gram-positive bacteria. These data show that these GS proteins are unusual multitasking proteins that function as enzymes and transcription regulators and unveil conserved mechanisms of activation of these enzymes and modes of GlnR binding to DNA. The findings also reveal unexpected features of this unique metabolic regulatory circuitry. First, our data indicate that the FBI-GS-GlnR interaction facilitates dimer formation of the weak GlnR dimer on the DNA by the close binding of two GlnR C-tails within each GS active site. Second, we showed that the FBI-GS-GlnR interaction also impacts GS function by stabilization of the GS inactive form. Third, our work breaks the current dogma that bacterial GS always form dodecamers. Finally, these studies also provide a template for the development of specific therapeutics, modeled from the GlnR-FBI-GS interaction, against Gram-positive pathogens.

## Methods

### Protein purifications

The genes encoding *Bs* GS, *Sa* GS, *Lm* GS, *Pp* GS, *Sa* full length (FL) GlnR, *Lm* FL GlnR, *Pp* FL GlnR*, Sa* GlnR(1-87), *Lm* GlnR(1-87), and *Pp* GlnR(1-87) were purchased from Genscript Corporation and subcloned into pET15b such that an N-terminal His-tag was expressed on each protein for purification (Piscataway, NJ, USA; http://www.genscript.com). *Escherichia coli* C41(DE3) cells were transformed with these expression vectors. Cells with each expression construct were grown at 37 °C in an LB medium with 0.10 mg/mL ampicillin to an OD_600_ of 0.3–0.4, then induced with 0.50 mM isopropyl β-d-thiogalactopyranoside (IPTG) at 15 °C overnight. Cells were harvested by centrifugation and then resuspended in Buffer A (50 mM Tris-Cl pH 7.5, 300 mM NaCl, 5% (v/v) glycerol, 5 mM MgCl_2_, 0.5 mM β-mercaptoethanol (βME), with 1X protease inhibitor cocktail and DNase I (~10 μL of 100 mg/mL DNase I per reconstitution). The resuspended cells were then disrupted with either a sonicator or microfluidizer and cell debris was removed by centrifugation (18,600 × g, 4 °C, 60 min). For each protein, the supernatant was loaded onto a cobalt NTA column. The column was washed with 300–500 mL of 2 mM imidazole in Buffer A and eluted in steps with 5, 10, 20, 30, 40, 50, 100, 200, 300 mM, and 1 M imidazole in Buffer A. Fractions were analyzed by SDS-PAGE and those containing the protein were combined. The His-tags were removed in all the proteins used in crystallography experiments by thrombin digestion overnight at room temperature (rt) using a thrombin cleavage capture kit (Novagen). Following His-tag removal, the proteins were put over a Ni-NTA column to remove the cleaved His-tag and uncleaved protein. Streptavidin agarose was added to remove biotinylated thrombin and the mixture spun to remove the beads. The GS proteins were concentrated using centrifugal ultrafiltration devices (centricons) with a 50 kDa MWCO (Millipore) and the GlnR proteins were concentrated using centricons with a 10 kDa MWCO (Millipore).

### Isothermal titration calorimetry (ITC) binding of GS to glutamine

ITC experiments were performed using a VP-ITC system (MicroCal Inc., Northampton, MA, USA) to analyze glutamine binding to GS. The *Sa* GS was used for the studies as it could be dialyzed and concentrated to high concentrations without noticeable precipitation. For ITC, the *Sa* GS sample was dialyzed into the ITC buffer (25 mM Tris pH, 7.5, 150 mM NaCl and 5 mM MgCl_2_) and the l-glutamine (l-glutamine is used in all studies and is denoted as glutamine) was dissolved in the same dialysis buffer. The ITC experiments were performed with glutamine (in the syringe) at the concentration of 50 mM and *Sa* GS at a hexamer concentration of 42 μM (in the sample cell). Glutamine was titrated into the sample cell containing *Sa* GS at 25 °C, and the resulting thermogram was fitted with Origin version 7.0 (MicroCal LLC).

### Glutamine synthetase (GS) enzyme assay

To interrogate GS enzymatic activity, we utilized the Biovision colorimetric GS activity kit (Cat K2056-100). In this sensitive assay, the ADP generated from GS activity is utilized in a subsequent reaction in the presence of an ADP converter, developer mix, and ADP probe to generate a colorimetric product read at an absorbance of OD_570_. For these assays, the *Sa, Pp*, and *Lm* GS proteins were the first buffer exchanged into the GS Assay Buffer from the kit. The protocol included with the kit was used for the assays and the absorbance was measured immediately at 570 nm using a Molecular Devices SpectraMax M5, after reaction initiation. In these experiments, *Pp* and *Sa* GS were present at 300 pM and the *Lm* GS at 600 pM. GlnR was added at a concentration of 10 nM. The measurements were done in kinetic mode at rt at 5 min intervals. One unit of GS activity was defined as the amount of enzyme that produces 1 µmol of ADP per min at pH 7.2 at 37 °C. The sample size for *Sa, Lm*, and *Pp* were 6, 4, and 3, respectively, and performed as independent experiments (on different days). To analyze the data, we performed Two-way ANOVA using the software GraphPad Prism 9.0.0. The two independent factors were GS and GlnR for each bacterial species. The *P* value for all the source of variation (interaction, row factor, and column factor—as specified in the software) are statistically significant (<0.0001) in all three cases (*Sa, Lm*, and *Pp*). The error bars represent the standard error of the mean.

### Crystallization and structure determinations of *Sa, Lm*, and *Pp* GS-Met-Sox-P-ADP transition state complex

For crystallization, *Sa* GS was concentrated to 30 mg/mL, *Pp* GS, to 15 mg/mL, and *Lm* GS, to 8 mg/mL All crystallizations were performed at rt by the hanging drop vapor diffusion method. The GS-Met-Sox-P-ADP transition state complexes were produced by mixing the GS proteins with 5 mM MgCl_2_, 5 mM ATP, and 5 mM Met-Sox for 1 h prior to setup. For crystallization of the *Sa* GS-Met-Sox-P-ADP complex, the protein solution was mixed 1:1 with a crystallization reagent composed of 28% (v/v) PEG 400, 0.1 M N-(2-Hydroxyethyl)piperazine-N´-(2-ethanesulfonic) acid (Hepes) pH 7.5, 0.2 M CaCl_2_. The crystals were cryopreserved straight from the drop. For crystallization of the *Pp* GS-Met-Sox-P-ADP complex, the reacted protein solution was mixed 1:2 with a crystallization reagent consisting of 30% (w/v) PEG monomethyl ether 2000, 0.1 M Tris pH 8.0. The crystals were cryopreserved by dipping them in a solution consisting of the crystallization reagent supplemented with 20% (v/v) glycerol before plunging into liquid nitrogen. For crystallization of the *Lm* GS-Met-Sox-P-ADP complex, the protein solution was mixed 1:1 with 15% (w/v) PEG 4000, 40 mM potassium phosphate dibasic pH 7.5, 20% (v/v) glycerol. The crystals were cryopreserved straight from the drop. Data were collected at ALS beamline 5.0.1 and processed with XDS^64^ (Version January 10, 2022) (Supplementary Table 3). The *Sa* GS-Met-Sox-P-ADP structure was solved by molecular replacement (MR) using a hexamer of the *Bs* GS transition state structure (PDB: 4LNI) as a search model in Phenix (version 1.19)^65^. Crystallographic symmetry generates a dodecamer from the hexamer. After an initial round of refinement in Phenix^65^, and replacement of *Sa* side chains, clear density was observed for the Met-Sox-P generated from GS catalysis along with ADP. The *Lm* GS-Met-Sox-P-ADP complex was solved by MR using the dodecamer *Sa* GS transition state structure as a search model. Molprobity (version 4.5.1) was used to check and validate every structure. The *Pp* GS TS structure was solved using a hexamer of the *Sa* GS TS. See Supplementary Table 3 for data collection and refinement statistics for the GS-Met-Sox-P-ADP structures.

### Crystallization and structure determination of *Sa* and *Lm* GlnR-DNA complexes

Crystals of the FL *Sa* and *Lm* GlnR-DNA complexes could not be obtained (FL *Pp* GlnR could not be concentrated sufficiently for crystallization trials). These GlnR proteins were subject to proteolysis over time perhaps impacting crystallization. Because our previous *Bs* GlnR-DNA crystals^39^, which were obtained with the FL *Bs* GlnR protein, revealed only residues GlnR 1-84, we used sequence alignments to determine truncation sites for the *Lm, Pp*, and *Sa* proteins, and generated the expression constructs encoding *Lm* GlnR(1-87), *Pp* GlnR(1-87), and *Sa* GlnR(1-87). The *Pp* GlnR(1-87) protein could also not be concentrated sufficiently for crystallization attempts. Hence, the *Lm* GlnR(1-87) and *Sa* GlnR(1-87) proteins were used in crystallization trials with DNA sites of different lengths and with different overhangs containing the GlnR binding operator site. Crystals of the *Sa* GlnR(1-87)-DNA complex were obtained by mixing the GlnR protein at 15 mg/mL at a molar ratio of 1:1.5 (protein:DNA) with a 21-mer operator site (top strand: 5′-CGTGTCAGATAATCTGACACG-3′) and mixing the protein-DNA complex 1:1 with a solution consisting of 50 mM sodium cacodylate pH 6.5, 13% (w/v) PEG 8000, 0.15 M calcium acetate, 20% (v/v) glycerol. Crystals grew in 5 days to a week and could be cryopreserved from the drop. Crystals of the *Lm* GlnR-DNA complex were produced by mixing *Lm* GlnR(1-87) at 5 mg/mL at a molar ratio of 1:1.5 (protein:DNA) with the 21-mer site used for crystallization with *Sa* GlnR(1-87) and mixing this solution 1:1 with 50 mM sodium cacodylate pH 6.5, 35% (v/v) MPD, 10 mM MgCl_2_. Small crystals were produced that diffracted weakly. To get crystals large enough for structure determination, the protein-DNA complex was concentrated two-fold just prior to crystallization setups and mixed with the same crystallization condition except the MPD concentration was reduced to 28% (v/v) to produce the largest crystals. These crystals could be cryopreserved from the drop. X-ray intensity data for both crystals were collected at ALS beamline 5.0.2 and the data were processed with XDS^64^.

The *Sa* GlnR-DNA complex structure was solved by molecular replacement (MR) using the *Bs* GlnR-DNA complex (PDB: 4R4E) as a search model with MOLREP^66^. Refinement was carried out in Phenix^65^. After the first round of refinement, the *Sa* side chains were substituted for the *Bs* side chains using Coot^67^ and refinement commenced. GlnR residues 6-83 were visible in the structure as well as all 21 nucleotides of each DNA duplex. In the final round of refinement, solvent molecules were added. The structure contains two GlnR dimer-DNA duplexes in the crystallographic asymmetric unit (ASU); the GlnR subunits in the complexes can be superimposed with rmsds of 0.25 Å (for 77 Cα atoms). See Supplementary Table 4 for data collection and refinement statistics. A *Sa* GlnR monomer bound to a half-site 21-mer was used in MR to solve the *Lm* GlnR-DNA structure. Crystallographic symmetry generates the GlnR dimer and duplex DNA in this crystal. After one round of refinement, the *Lm* residues were substituted. All the nucleotides of the DNA site in the ASU were visible in the structure but only GlnR residues 2–73 could be traced in the density. Molprobity (version 4.5.1) was used to check and validate the GlnR-DNA structures. The *Lm* GlnR-DNA crystals were highly anisotropic, possibly explaining the slightly elevated R values (Supplementary Table 4).

### Fluorescence polarization (FP) binding experiments

To measure DNA binding to *Sa* FL GlnR and GlnR(1-87), a fluoresceinated version of the 21-mer DNA site used for crystallization was obtained. For the experiment, increasing concentrations of the proteins were titrated into the sample cell containing 1 nM of the DNA in a buffer of 25 mM Tris pH 7.5, 100 mM NaCl, and 5 mM MgCl_2_. This same *Sa* GlnR(1-87) and buffer system was used in FP analyses to compare its DNA-binding affinity with the *Sa* GlnR(1-87)K48R and *Sa* GlnR(1-87)K48H mutant proteins. The mutant proteins were produced as artificial genes optimized for expression in *E. coli* and cloned into pET15b. To analyze *Sa* GlnR C-tail peptide binding to *Sa* GlnR(1-87) an N-terminal fluoresceinated GlnR C-tail peptide (the same as used for structural studies) was obtained from Genscript. In these experiments, increasing concentrations of *Sa* GlnR(1-87) were titrated into a sample cell containing 1 nM of the fluoresceinated peptide. Similarly, to measure binding affinities of FBI-GS proteins for their corresponding GlnR C-tails, fluoresceinated GlnR C-tails were used. The buffer for these experiments contained 25 mM Tris pH 7.5, 100 mM NaCl, 5 mM MgCl_2_, 20 mM glutamine. The resultant data were plotted using KaleidaGraph Version 4.5 for Mac; serial # 8011073 (Synergy Software) and the curves fit to deduce binding affinities. Three technical repeats were performed for each curve.

### Negative stain EM

For the negative stain EM experiments, purified *Bs* GS was in a buffer consisting of 25 mM Tris pH 7.5, 200 mM NaCl, 5% (v/v) glycerol, and 1 mM MgCl_2_. The *Bs* GS-TnrA and *Bs* GS-GlnR samples were in a buffer consisting of 25 mM Tris pH 7.5, 200 mM NaCl, 5% (v/v) glycerol, 1 mM MgCl_2_, 5 mM glutamine. *Bs* GS was mixed 1:1 stoichiometrically with FL TnrA and FL GlnR to generate the GS-TnrA and GS-GlnR sample complexes. Samples were diluted to a final concentration of 20–50 μg/mL and prepared for negative stain using sample buffer instead of water for the washes. Negatively stained specimens were imaged in a Tecnai 12 electron microscope (FEI Company) equipped with a Lab6 electron source and operated at 120 kV^68^. Micrographs were automatically collected under low-dose conditions using EPU (FEI Company) at a nominal magnification of 67,000×. Underfocused images (1–3 μm) were recorded on a US4000 CCD camera (Gatan) with a pixel size at the specimen level of 1.77 Å. Images were processed with EMAN2 (2.91)^69^ to produce 2D classes.

### Cryo-EM sample and grid preparation

Cryo-EM flow diagrams for processing and final structure analyses are provided in Fig. 2 and Supplementary Figs 1–4.

#### Bs GS14-Q-GlnR peptide

Purified *Bs* GS was buffer exchanged into Buffer B (12.5 mM Tris pH 7.5, 20 mM glutamine, 5 mM MgCl_2_, 150 mM NaCl, 2.5% (v/v) glycerol, 1 mM BME) using a 10 kDa MWCO spin filter (Millipore). Next, GS (1.0 mg/mL) was mixed with 2.5 mM *Bs* GlnR C-tail peptide (Genscript; sequence: MQAGRFQRGNTFRQGDMSRFFH) and incubated at rt for 30 min. For grid preparation, UltrAufoil R1.2/1.3 Au 300 (Quantifoil) holey gold grids were cleaned for 180 s using a PELCO easiGlow glow discharge cleaning system and 3 μL of the sample were applied at 95% humidity and 22 °C. Following a 10 s incubation period, the grids were blotted for 1.5 s and plunged frozen into liquid ethane using a Leica EM GP2 (Leica Microsystems). All grids were stored in liquid nitrogen until imaging.

#### Lm GS14-Q-GlnR peptide

Purified *Lm* GS was exchanged into Buffer B using a 10 kDa MWCO spin filter (Millipore). Next, *Lm* GS (0.9 mg/mL) was mixed with 2.5 mM *Lm* GlnR C-tail peptide (Genscript; sequence: QQAGRFVKQDATGKQQLPRF) and incubated at rt for 30 min. For grid preparation, Quantifoil R1.2/1.3 Cu 300 (Quantifoil) holey carbon grids were cleaned for 100 s using a PELCO easiGlow glow discharge cleaning system and 3 μL of the sample were applied at 95% humidity and 22 °C. Following a 10 s incubation period, the grids were blotted for 1.5 s and plunged frozen into liquid ethane using a Leica EM GP2 (Leica Microsystems).

#### Pp GS12-Q-GlnR peptide and Pp GS14-Q-GlnR peptide

Purified *Pp* GS was exchanged into Buffer B using a 10 kDa MWCO spin filter (Millipore). Next, *Pp* GS (0.8 mg/mL) was mixed with 2.5 mM *Pp* GlnR C-tail peptide (Genscript; sequence: KRPGQVSLIQGELSRFFNNR) and incubated at rt for 30 min. For grid preparation, Quantifoil R1.2/1.3 Cu 300 (Quantifoil) holey carbon grids were cleaned for 100 s using a PELCO easiGlow glow discharge cleaning system and 3 μL of the sample were applied at 95% humidity and 22 °C. Following a 10 s incubation period, the grids were blotted for 1.5 s and plunge-frozen into liquid ethane using a Leica EM GP2 (Leica Microsystems).

#### Sa GS12-Q-GlnR peptide

Purified *Sa* GS was buffer exchanged into Buffer B using a 10 kDa MWCO spin filter (Millipore). Next, GS (0.75 mg/mL) was mixed with 5 mM *Sa* GlnR C-tail peptide (Genscript; sequence: KPIGETLPINRGDLSRFIK) and incubated at rt for 30 min. For grid preparation, UltrAufoil R1.2/1.3 Au 300 (Quantifoil) holey gold grids were cleaned for 180 s using a PELCO easiGlow glow discharge cleaning system and 3 μL of the sample were applied at 95% humidity and 22 °C. Following a 10 s incubation period, the grids were blotted for 1.5 s and plunge-frozen into liquid ethane using a Leica EM GP2 (Leica Microsystems).

#### Lm GS12 apo

Purified *Lm* GS was exchanged into Buffer B using a 10 kDa MWCO spin filter (Millipore) and concentrated to 0.9 mg/mL. For grid preparation, Quantifoil R1.2/1.3 Cu 300 (Quantifoil) holey carbon grids were cleaned for 100 s using a PELCO easiGlow glow discharge cleaning system and 3 μL of the sample were applied at 95% humidity and 22 °C. Following a 10 s incubation period, the grids were blotted for 1.5 s and plunge-frozen into liquid ethane using a Leica EM GP2 (Leica Microsystems).

#### Pp GS12 apo

Purified *Pp* GS was exchanged into Buffer B using a 10 kDa MWCO spin filter (Millipore) and concentrated to 0.9 mg/mL. For grid preparation, Quantifoil R1.2/1.3 Cu 300 (Quantifoil) holey carbon grids were cleaned for 100 s using a PELCO easiGlow glow discharge cleaning system and 3 μL of the sample were applied at 95% humidity and 22 °C. Following a 10 s incubation period, the grids were blotted for 1.5 s and plunge-frozen into liquid ethane using a Leica EM GP2 (Leica Microsystems).

#### *Sa* GS12 apo

Purified *Sa* GS was exchanged into Buffer B using a 10 kDa MWCO spin filter (Millipore) and concentrated to 1.0 mg/mL. For grid preparation, UltrAufoil R1.2/1.3 Cu 300 (Quantifoil) holey gold grids were cleaned for 180 s using a PELCO easiGlow glow discharge cleaning system and 3 μL of the sample were applied at 95% humidity and 22 °C. Following a 10 s incubation period, the grids were blotted for 1.5 s and plunge-frozen into liquid ethane using a Leica EM GP2 (Leica Microsystems).

### Cryo-EM data collection

All cryo-EM data were collected on a 200 keV Thermo Fisher Scientific G3 Talos Arctica equipped with a Gatan K3 direct-electron detector. The microscope was aligned using a cross grating replica 2160 mm (TedPella) TEM grid and parallel illumination was obtained by adjusting the C2 lens in diffraction mode at 850 mm^70^. Coma Free alignment was done in SerialEM (version 3.8)^71,72^ and verified by acquiring 3 × 3 Zemlin Tableau^73^. Coma vs Image shift calibration was done in low-dose mode prior to data collection. Data were collected at a nominal magnification of 54,900 X at the detector level, corresponding to a pixel size of 0.88 Å. Data were collected via the beam-image shift method using an 11 × 11 or a 5 × 5 Multishot array in SerialEM^71,72^. The 70 µm condenser aperture and 100 µm objective aperture were inserted during data collection. A gain reference was collected in Digital Micrograph (Gatan) before starting data collection and a new dark reference was collected every 1 h during data collection, as implemented in the SerialEM data collection script. Movies were recorded as LZW compressed TIFF files and gain corrected in cryoSPARC Live (version 3.2)^74^.

### Cryo-EM image processing

All cryo-EM data were processed with cryoSPARC Live. Movies were gain corrected, motion and CTF corrected in real-time. Initial 2D classes were generated using Blob Picker on a subset of 50 micrographs. Classes with well-centered particles of interest and visible elements of secondary structure were used to pick particles with the Template Picker and generate an Ab-initio map with either D6 or D7 symmetry. Multiple rounds of 2D classification were used to aid in the centering and selection of particles. EM maps were generated by multiple rounds of Homogenous Refinement, Global and Local CTF Refinement until no improvement in resolution and Beta Factor were obtained. Exposures were split into separate optics groups by multishot position or processed with each micrograph as its own optic group. The number of micrographs, number of particles, box size, symmetry, resolution, and beta factor for each map is provided in Supplementary Tables 1, 2.

### Cryo-EM model building and refinement

To model the GS-peptide structures, first, one GS subunit and one peptide from the published *Bs* GS-Q-TnrA peptide crystal structure^39^ (PDB: 4S0R) were docked in the maps using UCSF Chimera X 11.3^75^. The GlnR peptide had to be rebuilt. GS and peptide residues were mutated to match the correct sequence using Coot. Multiple rounds of fitting in Coot^67^ and real-space refinement in Phenix^65^ were performed to improve the quality of the models. After fitting one subunit, multiple copies of the GS and peptide models were generated and docked into the maps to complete the dodecamer and tetradecamer complexes. This was followed by a final round of refinement in Phenix^65^ and fitting in Coot^67^. Apo GS structures were generated using the same workflow.

## Supplementary information

Supplementary Information

## Data Availability

The structural data referenced in this study can be found in the Protein Data Bank under the accession codes 4LNI, 4R4E, and 4S0R. The structural data generated in this study have been deposited in the Protein Data Bank under the accession codes 7TEA, 7TEC, 7TDP, 7TEN, and 7TDV for the crystal structures. The structural data generated in this study by cryo-EM have been deposited in the Protein Data Bank under the codes 7TF7, 7TFE, and 7TFD for the apo GS structures and 7TFC, 7TFB, 7TFA, 7TF6, and 7TF9 for the FBI-GS-GlnR structures.
