# Supplementary material for: Molecular dissection of the glutamine synthetase-GlnR nitrogen regulatory circuitry in Gram-positive bacteria
Source: Nat Commun. 2022 Jul 1;13:3793. doi: 10.1038/s41467-022-31573-0 (PMC9249791; doi:10.1038/s41467-022-31573-0)
Supplement: Supplementary file 1 — Supplementary Information [file 41467_2022_31573_MOESM1_ESM.pdf]

Supplementary Information

Molecular dissection of the Glutamine Synthetase-GlnR nitrogen regulatory circuitry in Gram-positive bacteria

M. A. Schumacher *et al.*

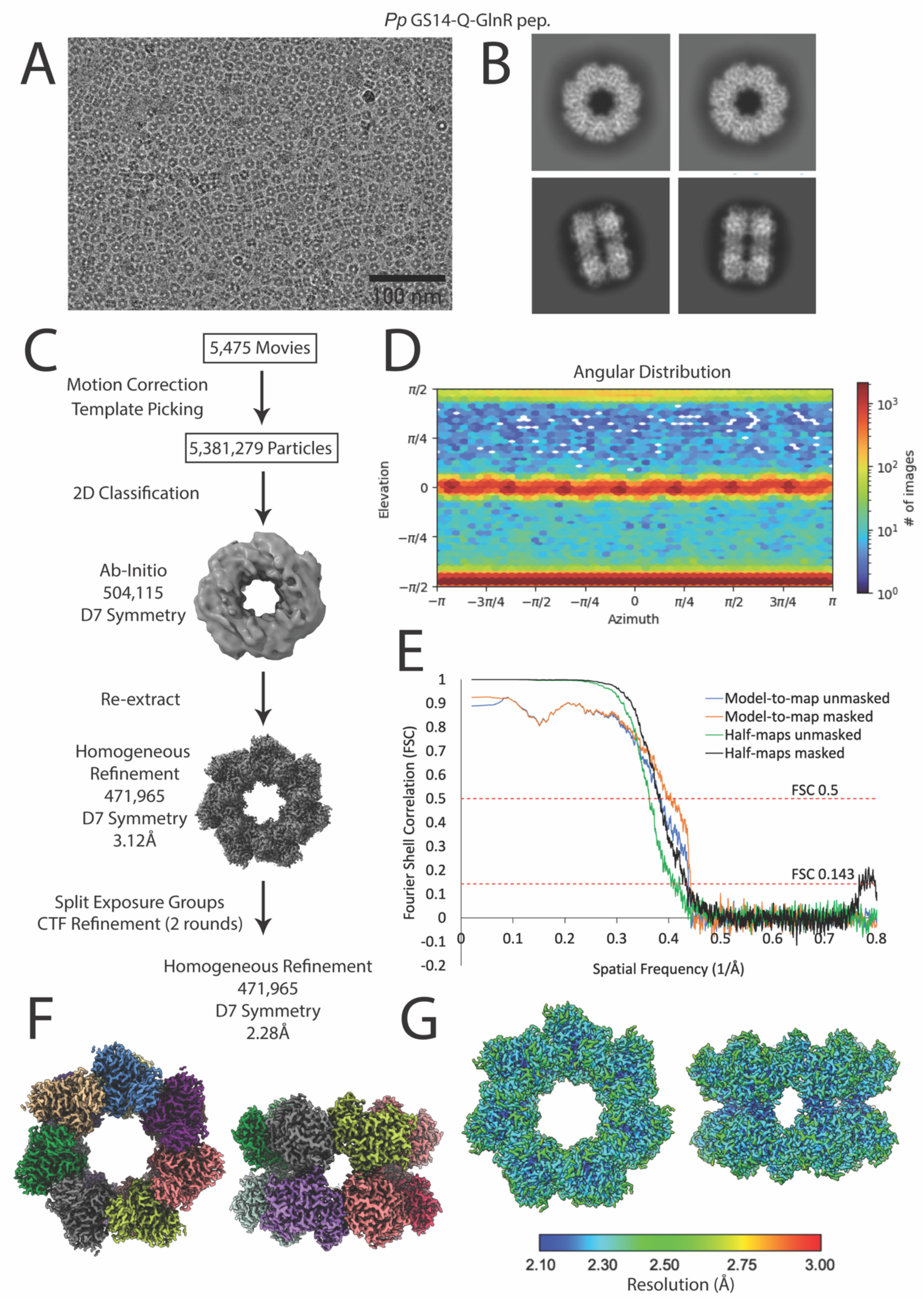

Supplementary Figure 1.

Cryo-EM data processing of the *Pp* GS14-Q-GlnR peptide dataset

(A) Representative micrograph used for processing of the *Pp* GS14-Q-GlnR peptide complex on a holey carbon grid. (**B)** A subset of the 2D classes showing top and side views of the tetradecameric complex. (**C)** Summary of the data processing workflow. (**D)** Angular distribution plot of the final particle set. (**E)** Masked and unmasked half-map and model-to-map FSC curves. (**F**) Final sharpened map colored by individual GS subunit. (**G)** GS structure colored according to local resolution, with blue to red representing high to low resolution.

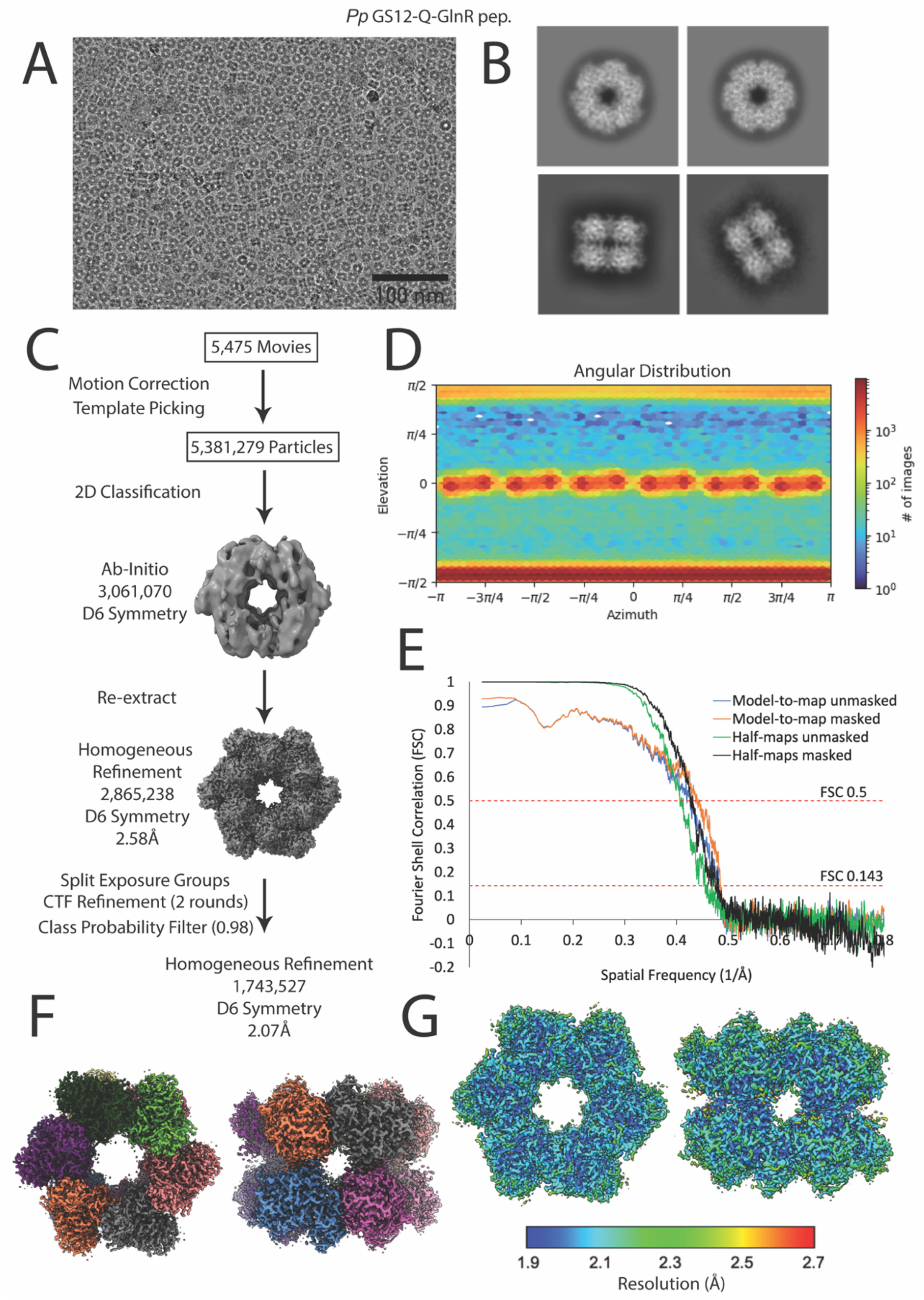

Supplementary Figure 2.
Cryo-EM data processing of the *Pp* GS12-Q-GlnR peptide dataset
(A) Representative micrograph used for data processing of *Pp* GS12-Q-GlnR peptide complex on a holey carbon grid. Note Panel A is the same as Figures S1A as the same data were used to acquire *Pp* GS12-Q-GlnR and *Pp* GS14-Q-GlnR complexes from the grids. (**B**) A subset of the 2D classes showing top and side views of the dodecameric complex. (**C**) Summary of the data processing workflow. (**D**) Angular distribution plot of the final particle set. (**E**) Masked and unmasked half-map and model-to-map FSC curves. (**F**) Final sharpened map colored by individual GS subunit. (**G**) GS structure colored according to local resolution, with blue to red representing high to low resolution

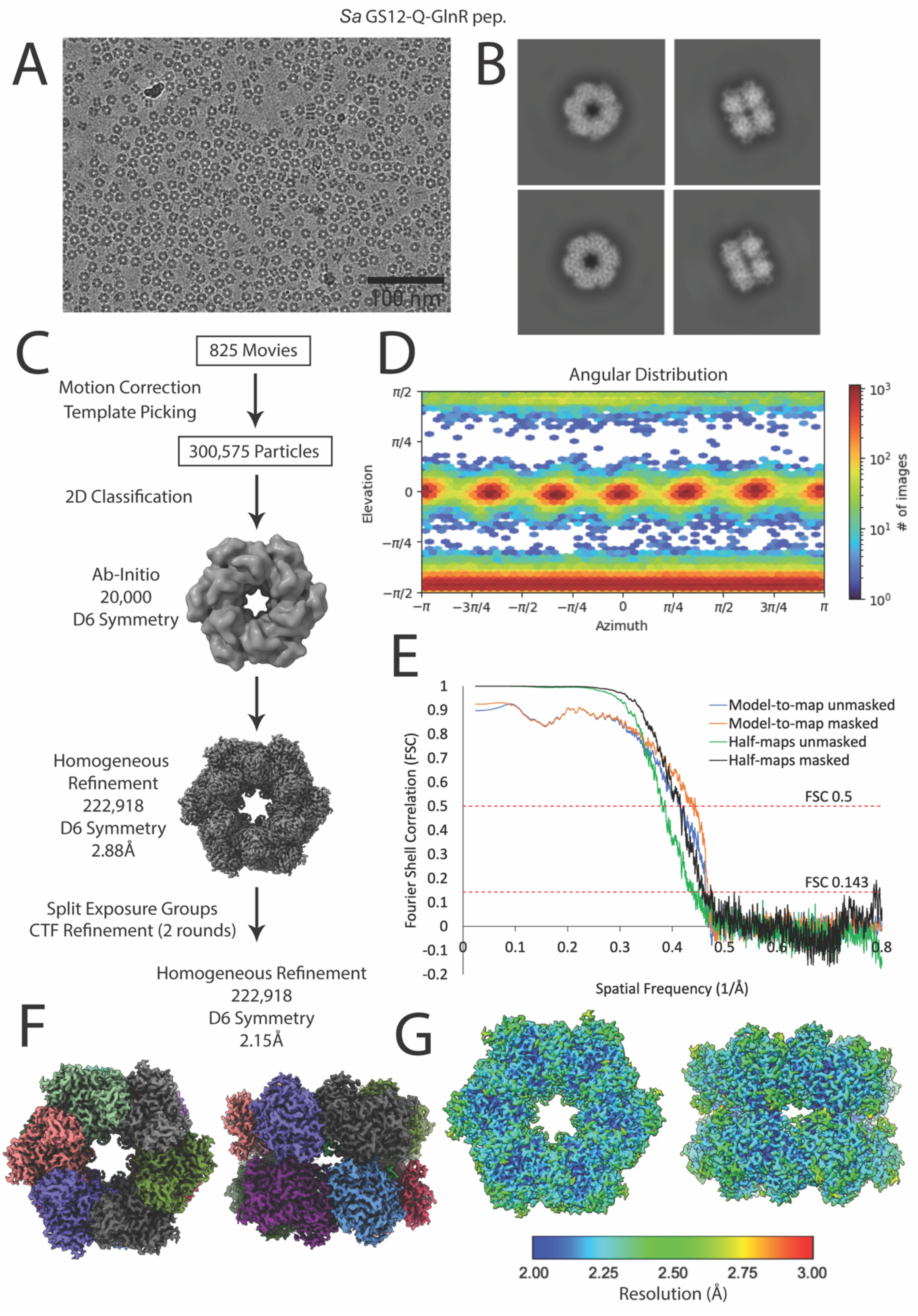

Supplementary Figure 3.
Cryo-EM data processing of the *Sa* GS12-Q-GlnR peptide dataset
(A) A representative micrograph of the *Sa* GS12-Q-GlnR pep. complex on a holey gold grid. (**B)** A subset of the 2D classes showing top and side views of the dodecameric complex. (**C)** Summary of the data processing workflow. (**D)** Angular distribution plot of the final particle set. (**E)** Masked and unmasked half-map and model-to-map FSC curves. (**F)** Final sharpened map colored by individual GS subunit. (**G)** GS structure colored according to local resolution, with blue to red representing high to low resolution.

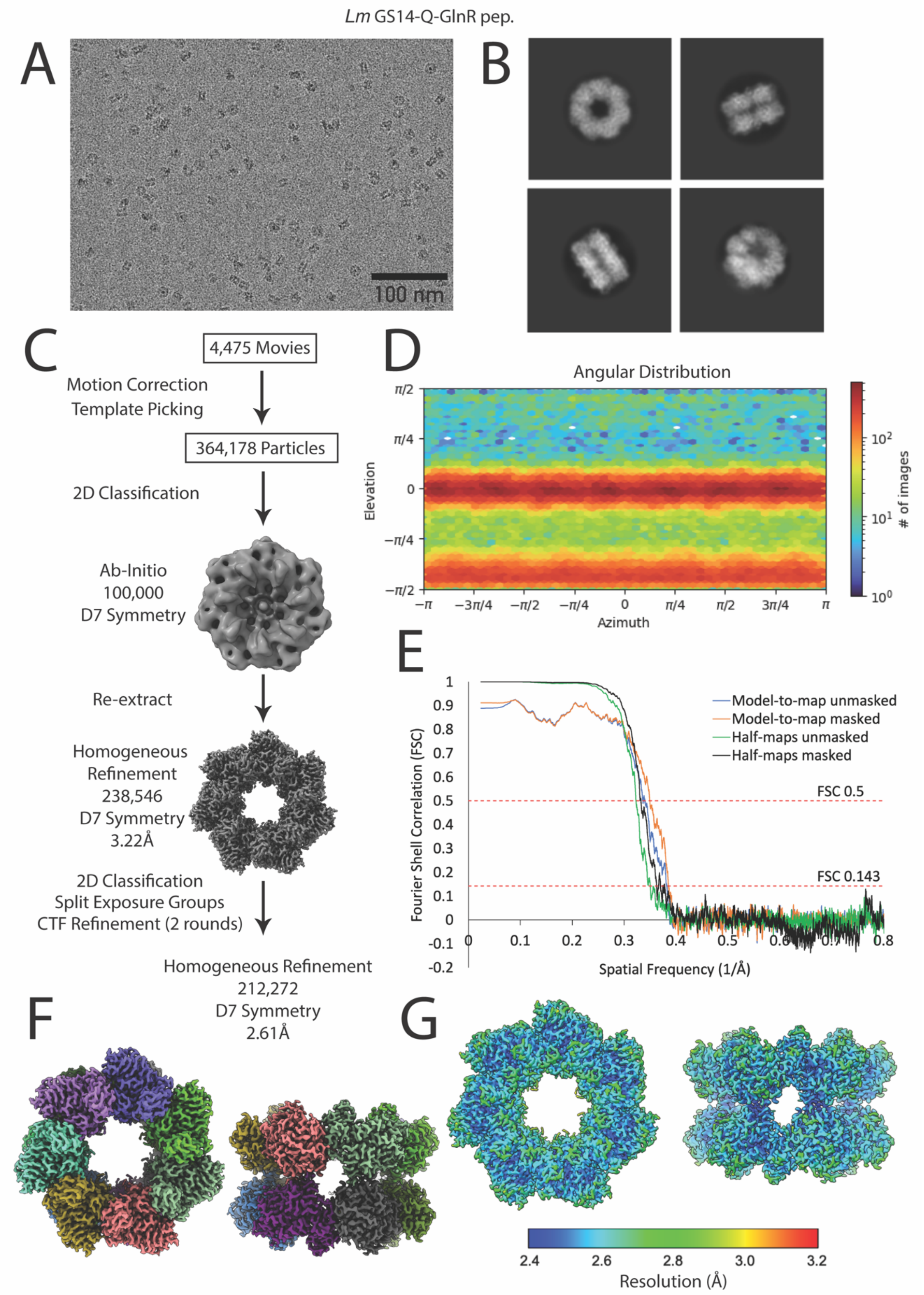

Supplementary Figure 4.

Cryo-EM data processing of the *Lm* GS14-Q-GlnR peptide dataset.

(A) A representative micrograph of the *Lm* GS14-Q-GlnR pep. complex on a holey carbon grid. (**B)** A subset of the 2D classes showing top and side views of the tetradecameric complex. (**C)** Summary of the data processing workflow. (**D)** Angular distribution plot of the final particle set. (**E)** Masked and unmasked half-map and model-to-map FSC curves. (**F)** Final sharpened map colored by individual GS subunit. (**G)** GS structure colored according to local resolution, with blue to red representing high to low resolution.

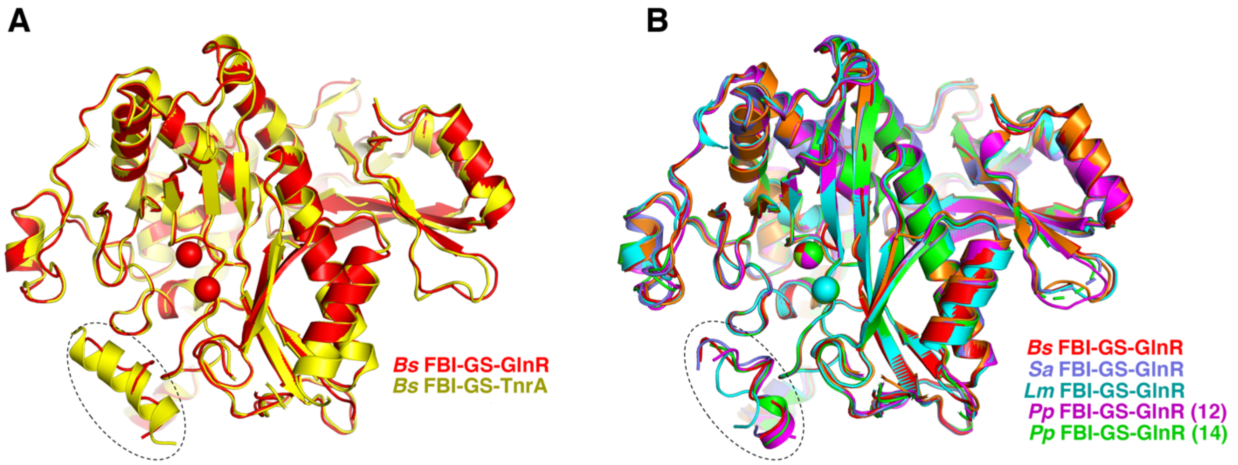

Supplementary Figure 5.
Superimpositions of GlnR and TnrA bound GS subunits.
(**A**) Overlay of *Bs* FBI-GS subunits bound to GlnR (red) and TnrA (yellow) showing overall similarity in subunit conformation and similar location of bound GlnR to TnrA peptide. The GlnR and TnrA peptides are circled.
(**B**) Overlay of the FBI-GS subunits in complex with GlnR showing that all bind the GlnR in similar locations (the GlnR peptides are circled). *Pp* FBI-GS-GlnR (12) and *Pp* FBI-GS-GlnR (14) correspond to the structures with dodecameric and tetradecameric *Pp* GS, respectively. The *Bs* FBI-GS-GlnR, *Sa* FBI-GS-GlnR, *Lm* FBI-GS-GlnR, *Pp* FBI-GS-GlnR (12) and *Pp* FBI-GS-GlnR (14) subunits are colored red, slate, cyan, magenta and green, respectively.

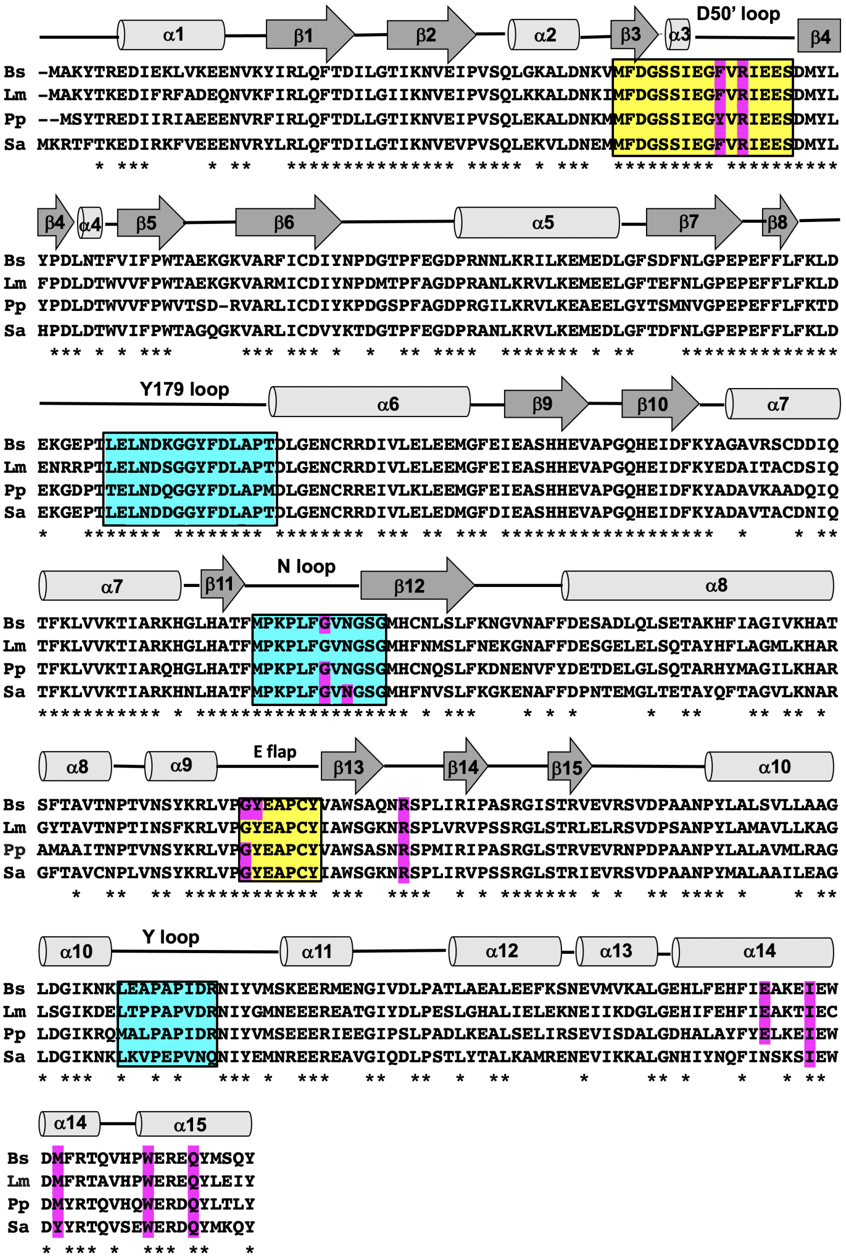

Supplementary Figure 6. Multiple sequence alignment of Gram-positive glutamine synthetases analyzed in this study.
Bs, Lm, Pp and *Sa* GS protein sequences are aligned. Asterisks under the sequence indicate residues that are conserved among all homologs. Secondary structural elements from the *Bs* GS structure (shared among the GS structures) are indicated over the sequences. The active site loops are labeled. The E flap and D50' loop are highlighted in yellow and the other active site loops, in cyan. Residues that contact GlnR are highlighted in magenta. The protein sequence entry IDs are *Bs* GS, P12425.3; *Sa* GS, WP_086038154.1; *Lm* GS, EAC9051058.1; *Pp* GS, WP_016822091.1.

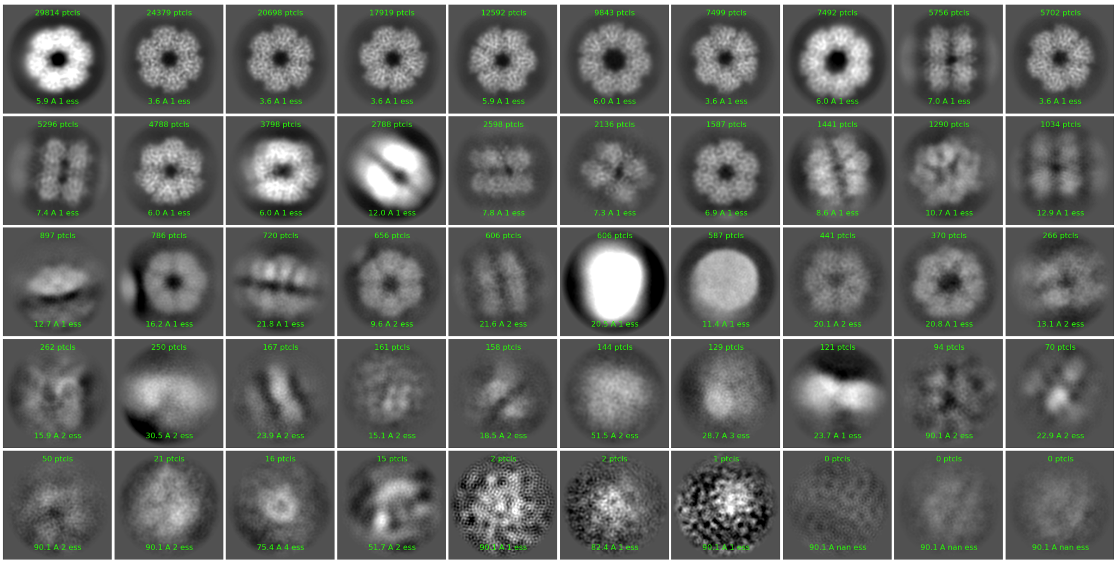

Supplementary Figure 7. 2-D classes of *Pp* apo GS. A subset of the 2D classes for the *Pp* apo GS structure showing top and side views of dodecameric and, though minor, some tetradecameric complexes.

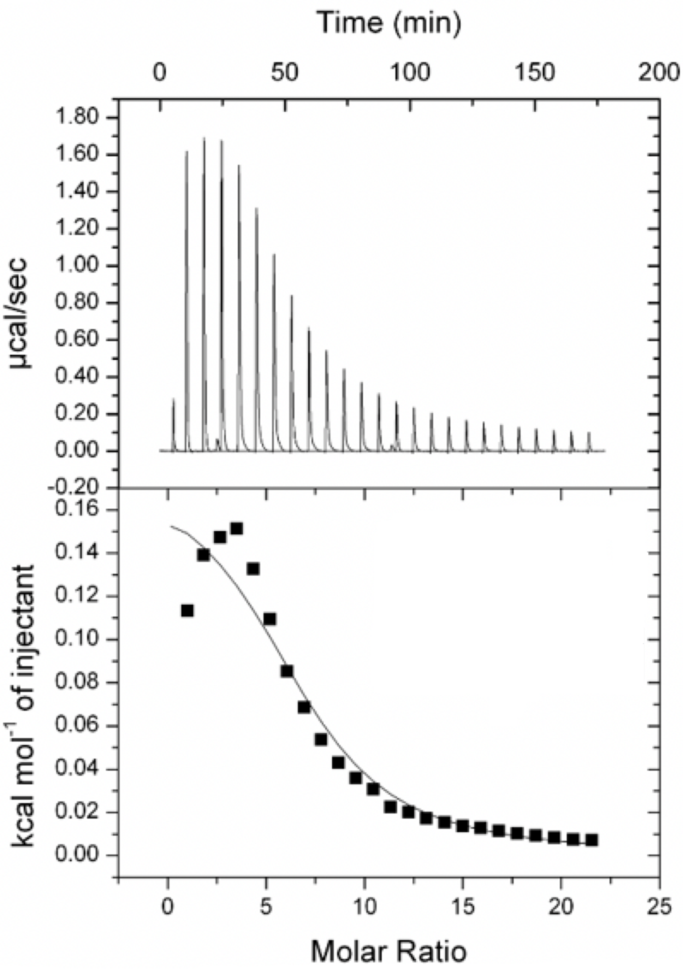

Supplementary Figure 8. Isothermal titration calorimetry of glutamine binding to *Sa* GS. Top show the experimental data and below, the fit. The resultant K_d is 0.5 +/- 0.09 mM. Source data are provided in the Source Data file.

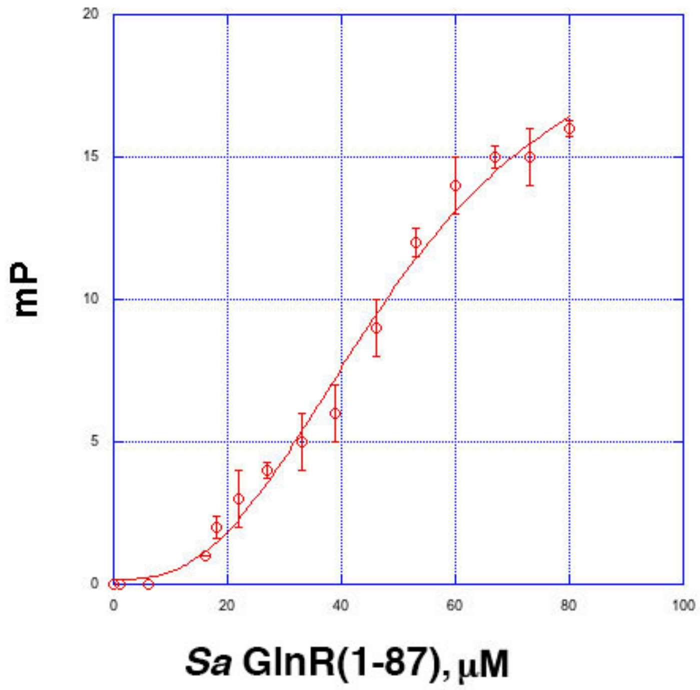

Supplementary Figure 9. The *Sa* GlnR C-tail binds to truncated *Sa* GlnR(1-87). Fluorescence polarization binding isotherm following the titration of *Sa* GlnR(1-87) into a reaction cell containing 1 nM fluoresceinated *Sa* GlnR C-tail. The y-axis is the change in millipolarization units (mP) and the x-axis shows *Sa* GlnR(1-87) concentration. The K_d is 56 ± 9 µM. Note, the binding affinity may be higher with addition of more of the GlnR C-tail (only the C-terminal 19 residues were included in the F-peptide). Shown are representative curves from three technical repeats. The error bars represent SD. Data are presented as mean values +/- SD. The source data are provided in the Source Data file.

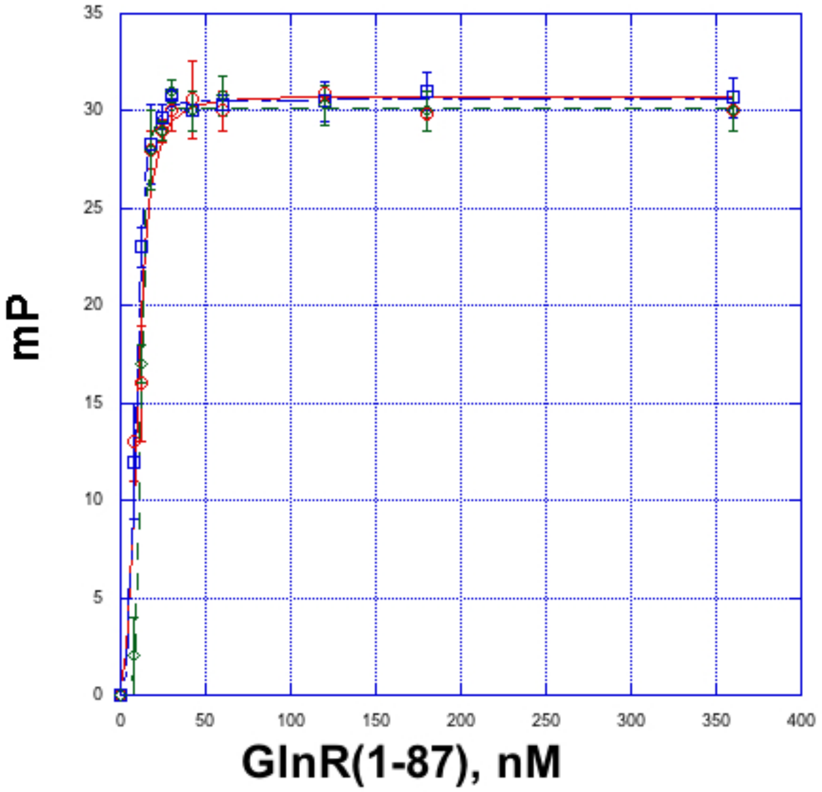

Supplementary Figure 10. The *Sa Sa* GlnR(1-87), *Sa* GlnR(1-87)K48R and *Sa* GlnR(1-87)K48H binding to operator DNA.
Fluorescence polarization binding isotherm following the titration of *Sa* GlnR(1-87) or *Sa* GlnR(1-87)K48R or *Sa* GlnR(1-87)K48H into a reaction cell containing 1 nM fluoresceinate d operator DNA (top strand: 5'-CGTGTCAGATAATCTGACACG-3'). The GlnR(1-87) WT binding isotherm is red, the GlnR(1-87)K48R is blue and the GlnR(1-87)K48H binding isotherm is green. The curves are representative curves from three technical repeats. The error bars represent SD. Data are presented as mean values +/- SD.
The source data are provided in the Source Data file.

Supplementary Table 1. Cryo-EM data collection, refinement, and validation statistics: GS-Q-GlnR peptide structures

	***Bs* GS14-Q-GlnR** PDB: 7TFC EMD-25869	***Pp* GS14-Q-GlnR** PDB: 7TFB EMD-25868	***Pp* GS12-Q-GlnR** PDB: 7TFA EMD-25867	***Sa* GS12-Q-GlnR** PDB: 7TF6 EMD-25863	***Lm* GS14-Q-GlnR** PDB: 7TF9 EMD-25866
**Data collection and Processing**					
Electron microscope	Talos Arctica G3 (UNC)	Talos Arctica G3 (UNC)	Talos Arctica G3 (UNC)	Talos Arctica G3 (UNC)	Talos Arctica G3 (UNC)
Detector	K3	K3	K3	K3	K3
Magnification	54,900	54,900	54,900	54,900	54,900
Voltage (keV)	200	200	200	200	200
Electron exposure (e-/Å^2)	43.74	46.27	46.27	50.5	42.65
Defocus range (μm)	0.4-2.2	0.5-2.1	0.5-2.1	0.5-2.2	0.3-2.2
Pixel size (Å)	0.88	0.88	0.88	0.88	0.88
Total extracted particles (no.)	4,589,551	5,381,279	5,381,279	300,575	364,178
Refined particles (no.)	1,910,475	471,965	2,865,238	222,918	238,546
Movies	5,328	5,475	5,475	825	4,475
**Reconstruction**					
Final particles (no.)	1,910,475	471,965	1,743,527	222,918	212,272
Symmetry imposed	D7	D7	D6	D6	D7
FSC 0.143 (unmasked/masked) (Å)	2.2/1.96	2.8/2.28	2.5/2.07	2.8/2.15	3.1/2.61
Map sharpening B-factor (Å^2)	74.9	78.8	69.1	62	110.5
**Refinement**					
**Model composition**					
Non-hydrogen atoms	50,302	49,476	42,504	43,140	49,714
Protein residues	6,356	6,328	5,424	5,400	6,286
Ions/Ligands	42	42	36	36	36
MolProbity score	1.82	1.99	1.80	1.59	2.22
Clash score	6	7	6	4	9
**Bonds (RMSD)**					
Bond lengths (Å)	0.003	0.005	0.003	0.002	0.005
Bond angles (°)	0.524	0.595	0.560	0.494	0.674
**Ramachandran plot**					
Favored (%)	95.88	94.85	96.36	96.29	95.95
Allowed (%)	4.12	5.07	3.62	3.71	4.05
Disallowed (%)	0.00	0.08	0.02	0.00	0.00

Supplementary Table 2. Cryo-EM data collection, refinement, and validation statistics: apo GS structures

	***Sa* GS12 apo**<br>PDB: 7TF7<br>EMD-25864	***Lm* GS12 apo**<br>PDB: 7TFE<br>EMD-25871	***Pp* GS12 apo**<br>PDB: 7TFD<br>EMD-25870
**Data collection and Processing**			
Electron microscope	Talos Arctica G3 (UNC)	Talos Arctica G3 (UNC)	Talos Arctica G3 (UNC)
Detector	K3	K3	K3
Magnification	54,900	54,900	54,900
Voltage (keV)	200	200	200
Electron exposure (e-/Å^2)	46.5	42.6	41.674
Defocus range (μm)	0.5-2.4	0.2-2.5	0.5-2.7
Pixel size (Å)	0.88	0.88	0.88
Total extracted particles (no.)	577,364	634,162	715,911
Refined particles (no.)	477,190	278,295	113,317
Movies	725	2,764	1,116
**Reconstruction**			
Final particles (no.)	474,190	260,267	105,664
Symmetry imposed	D6	D6	D6
FSC 0.143 (unmasked/masked) (Å)	2.7/2.13	3.4/2.85	3.9/3.16
Map sharpening B-factor (Å^2)	69.6	109.1	92
**Refinement**			
**Model composition**			
Non-hydrogen atoms	39,974	41,388	41,408
Protein residues	4,980	5,316	5,292
Ions/Ligands	0	24	24
MolProbity score	1.53	2.04	2.00
Clash score	6	8	13
**Bonds (RMSD)**			
Bond lengths (Å)	0.004	0.004	0.004
Bond angles (°)	0.615	0.675	0.707
**Ramachandran plot**			
Favored (%)	97.38	95.24	95.01
Allowed (%)	2.54	4.76	4.97
Disallowed (%)	0.08	0.00	0.02

Supplementary Table 3. Crystallographic data collection and refinement statistics: GS-Met-Sox-P-ADP structures

	Pp GS-Met-Sox-P-ADP	*Lm* GS-Met-Sox-P-ADP	*Sa* GS-Met-Sox-ADP
**Data collection**			
Pdb code	7TDP	7TEN	7TDV
Space group	$P4_22_12$	P1	$P4_12_12$
Cell dimensions			
a, *b*, *c* (Å)	164.0,164.0,141.5	112.6,137.6,138.0	154.6,154.6,299.3
α, β, γ (°)	90.0,90.0,90.0	60.7,87.2,68.4	90.0,90.0,90.0
Resolution (Å)	48.7 – 1.98 (2.05-1.98)*	63.9-3.50 (3.62-3.50)	48.8-2.92 (3.02-2.92)
R_{sym}	0.108 (1.154)	0.184 (1.150)	0.165 (1.780)
R_{pim}	0.030 (0.544)	0.130 (0.966)	0.049 (0.517)
$I / \sigma I$	15.4 (1.4)	4.8 (1.2)	19.1 (2.1)
Completeness (%)	98.5 (89.8)	74.7 (77.1)	99.9 (100.0)
Redundancy	13.1 (4.6)	3.1 (2.1)	28.6 (19.4)
CC(1/2)	0.999 (0.486)	0.995 (0.323)	0.999 (0.744)
**Refinement**			
Resolution (Å)	48.7-1.98	63.9-3.50	48.8-2.92
No. reflections	132443 (12010)	62204 (6451)	79309 (7783)
R_{work} / R_{free} (%)	17.2/20.2	20.0/27.2	20.0/25.8
No. atoms			
Protein	11485	41996	21817
Ligand/ion	135	504	278
Water	821	0	307
B-factors			
Protein	42.2	111.4	69.5
Ligand/ion	32.4	107.9	65.6
Water	46.1	-	64.5
R.m.s. deviations			
Bond lengths (Å)	0.007	0.005	0.011
Bond angles (°)	1.02	0.952	1.22
Ramachandran analyses			
Favored (%)	97.2	89.8	94.6
Disallowed (%)	0.0	0.17	0.0

*Values in parentheses are for highest-resolution shell.

Supplementary Table 4. Crystallographic data collection and refinement statistics: GlnR-DNA complexes

	Sa GlnR-DNA complex	*Lm* GlnR-DNA complex
**Data collection**		
Pdb code	7TEA	7TEC
Space group	$I2_12_12_1$	$P222_1$
Cell dimensions		
a, *b*, *c* (Å)	66.5,99.3,238.9	28.6,47.7,121.4
α, β, γ (°)	90.0,90.0,90.0	90.0,90.0,90.0
Resolution (Å)	43.06 – 2.35 (2.47-2.35)*	37.5-3.45 (3.58-3.45)
R_{sym}	0.042 (0.539)	0.064 (0.418)
R_{pim}	0.027 (0.487)	0.038 (0.235)
$I / \sigma I$	21.4 (1.9)	8.9 (2.0)
Completeness (%)	98.6 (91.4)	96.7 (92.7)
Redundancy	6.2 (5.7)	4.1 (4.2)
CC(1/2)	1.000 (0.818)	0.998 (0.710)
**Refinement**		
Resolution (Å)	43.06-2.35	37.5-3.45
No. reflections	28493 (2606)	2399 (237)
R_{work} / R_{free} (%)	22.5/26.4	27.1/30.6
No. atoms		
Macromolecule	4347	980
Ligand/ion	2	0
Water	23	0
B-factors		
Protein/DNA	73.8	73.3
Ligand/ion	63.4	-
Water	56.8	-
R.m.s. deviations		
Bond lengths (Å)	0.009	0.003
Bond angles (°)	1.12	0.60
Ramachandran analyses		
Favored (%)	94.3	94.3
Disallowed (%)	0.0	0.0

*Values in parentheses are for highest-resolution shell.
